# First‑line endocrine therapy for hormone receptor positive and HER‑2 negative metastatic breast cancer: A Bayesian network meta‑analysis

**DOI:** 10.3892/ol.2024.14646

**Published:** 2024-08-28

**Authors:** Yi-Cheng Jiang, Jing-Jing Yang, Hai-Tian Zhang, Rui Zhuo, Sebastian De La Roche, Luz Angela Torres-De La Roche, Rudy Leon De Wilde, Jie Dong

**Affiliations:** 1 Department of Breast Surgery, EUSOMA Certified Breast Center, Guilin Traditional Chinese Medicine Hospital of China, Guilin, Guangxi Zhuang Autonomous Region 541002, P.R. China; 2 Department of Neonates, Guilin People's Hospital, Guilin, Guangxi Zhuang Autonomous Region 541001, P.R. China; 3 Department of Breast Disease, Guangxi International Zhuang Medical Hospital, Nanning, Guangxi Zhuang Autonomous Region 530001, P.R. China; 4 International Max Planck Research School in Molecular Biology, Georg-August-University of Gottingen, D-37077 Gottingen, Germany; 5 Department of Gynecology, Pius Hospital, Carl von Ossietzky University, D-26121 Oldenburg, Germany

**Keywords:** metastatic breast cancer, endocrine therapy, hormone receptor positive, receptor tyrosine-protein kinase erbB-2-negative, Bayesian network meta-analysis

## Abstract

Endocrine therapy has become the fundamental treatment option for hormone receptor-positive (HR^+^) and receptor tyrosine-protein kinase erbB-2-negative (HER2^−^) metastatic breast cancer (mBC). While treatments incorporating cyclin-dependent kinase (CDK)4 and 6 inhibitors are more prevalent than ever, comparisons among those regimens are scarce. The aim of the present study was to identify the most effective maintenance treatment for patients with HR^+^ and HER2^−^ mBC. To this end, databases including PubMed, Embase, Cochrane Library, Scopus and Google Scholar were searched from inception to August, 2023. The endpoints comprised overall survival (OS) and progression free survival (PFS). For dichotomous variants, hazard ratios (HRs) and odds ratios (ORs) were generated, while standard mean difference (SMD) was used for consecutive variants by Bayesian network meta-analysis to make pairwise comparisons among regimens, to determine the optimal therapy. These processes were conducted using Rstudio 4.2.2 orchestrated with STATA 17.0 MP. A total of 16 randomized controlled trials including 7,174 patients with 11 interventions were analyzed. Compared with aromatase inhibitor (AI), palbociclib plus AI (PalboAI) exhibited a significantly longer PFS up to the 36th month of follow-up [HR=1.7; 95% credible interval, 1.36–2.16], including on the 3rd [OR=2.22; 95% confidence interval (CI), 1.10–4.47], 6th (OR=2.39; 95% CI, 1.21–4.69), 12th (OR=1.94; 95% CI, 1.34–2.79), 18th (OR=2.38; 95% CI, 1.65–3.44), 24th (OR=2.39; 95% CI, 1.67–3.43), 30th (OR=2.10; 95% CI, 1.62–2.74) and 36th (OR=2.66; 95% CI, 1.37–5.18) month of follow-up. Additionally, abemaciclib plus fulvestrant exhibited significant effects compared with AI alone between 12 and 36 months. Ribociclib plus fulvestrant, ribociclib plus AI and dalpiciclib plus AI exerted significant effects compared with AI alone between 12 and 30 months. Considering the effect on OS and PFS together with adverse reactions, safety, medical compliance and route of administration, PalboAI was found to be the optimal treatment for HR^+^/HER2^−^mBC. However, additional head-to-head clinical trials are warranted to confirm these findings.

## Introduction

The lifetime risk of breast cancer (BC) for women has increased over the last four decades, making it the most common malignancy and the second leading cause of cancer-related mortality in women (1). BC places an enormous burden on the families of patients and national health systems (2). During 2023, 297,790 cases of BC were newly diagnosed in the United States, resulting in 43,170 deaths (1). Although the overall death rate has declined, black American women are at a higher risk of death compared with white American women (27.6 vs. 19.7 deaths per 100,000 in 2016–2020), as are adult women >50 years (12.1 vs. 6.5 deaths per 100,000 in 2016–2020) (1). The risk of local and metastatic recurrence is 5–60%, which can occur more than three decades after the primary diagnosis. In total, >70% of patients with metastatic BC (mBC) have hormone receptor positive (HR^+^) and receptor tyrosine-protein kinase erbB-2-negative (HER2^−^) disease (3). Endocrine therapy is recommended for these patients to improve their chances of survival, according to oncology guidelines (4). However, there is currently a lack of evidence on the most effective regimen for the maintenance treatment of patients with HR^+^/HER2^−^ mBC.

Historically, limited by less advanced medical science, tamoxifen and medroxyprogesterone alone were standard treatment options for patients with HR^+^/HER2^−^ mBC (5). The emergence of aromatase inhibitors (AIs), including letrozole and fulvestrant, marked an important milestone in endocrine therapy for mBC, due to their powerful antitumor effect (6). Fulvestrant was set as the standard treatment method in authoritative guidelines, paving the way for setting endocrine therapy as the principal remedy in these patients (7,8). Further research, including the PALOMA-1 trial, reported a longer progression-free survival (PFS) when a cyclin-dependent kinase (CDK)4/6 inhibitor, namely palbociclib, was added to fulvestrant to treat patients who had disease progression during endocrine therapy, starting a new era in the treatment of HR^+^/HER2^−^ mBC (9). This led to the development of several CDK4/6 inhibitors, including abemaciclib, dalpiciclib and ribociclib, which have been used in various trials evaluating the combined efficacy of CDK4/6 inhibitors plus endocrine therapy, including fulvestrant or other AIs, with encouraging results (10). Although clinicians are fortunate to have a number of strategies in the treatment of HR^+^/HER2^−^ mBC, their decisions are based on few head-to-head comparisons between regimens.

In recent years, there has been an increasing number of Bayesian network meta-analyses (NMAs) on CDK4/6 inhibitors, with non-conclusive evidence. In 2017, Chirila *et al* (11) reported that, compared with other endocrine therapies in untreated patients with advanced/mBC, the palbociclib plus AI regimen led to a significant increase in PFS. Similarly, in 2020, Liu *et al* (12) showed that palbociclib plus fulvestrant was the most effective treatment, but those results were limited since the authors included some second-line drug studies in the analysis, such as studies on everolimus. In terms of data analytics, the authors only considered the covariates of hazard ratio (HR) and inferred the result by surface under the cumulative ranking curve (SUCRA), rendering the final results less reliable.

For that reason, the present Bayesian NMA of first-line randomized controlled trials (RCT) included direct and indirect comparisons among regimens to identify the most effective maintenance treatment for patients with HR^+^/HER2^−^ mBC that, in turn, could improve clinical oncology.

## Materials and methods

The present Bayesian NMA was guided by the Preferred Reporting Items for Systematic Reviews and Meta-analysis guidelines (13).

### Search strategy

Google Scholar (https://scholar.google.hk), Cochrane Library (Cochrane Central Register of Controlled Trials; CENTRAL (https://www.cochranelibrary.com), PubMed (https://pubmed.ncbi.nlm.nih.gov), Scopus (https://www.scopus.com) and Embase (https://www.embase.com) were searched from inception to August, 2023, with the following MeSH terms: (‘Breast Cancer’ OR ‘Breast Carcinoma’ OR ‘Breast Neoplasm’ OR ‘Breast Tumor’ OR ‘Breast Malignant Tumor’) AND (‘Metastatic’ OR ‘Advanced’) AND (‘First-Line’ OR ‘First Line’ OR ‘Initial’) AND (‘Hormone Receptor Positive’ OR ‘Endocrine Receptor Positive’ OR ‘Endocrine Sensitivity’) AND (‘HER-2 Negative’ OR ‘Human Epidermal Factor Receptor 2 Negative’ OR ‘ErbB-2 Receptor Negative’ OR ‘C-erbB-2 Negative’) AND (‘Randomized’ OR ‘Allocation Random’ OR ‘Randomization’).

### Selection criteria

The study inclusion criteria were as follows: i) The study subjects had been diagnosed with HR^+^/HER2^−^ mBC; ii) the study was an RCT regarding first-line endocrine therapy; and iii) sufficient information was provided on the PFS and/or overall survival (OS). The exclusion criteria were as follows: i) The data required for analysis was not reported; ii) articles were observational studies, letters or reviews; and iii) the articles were not written in English.

### Data extraction and quality assessment

In total, two investigators independently searched and assessed the eligibility of each study by reading the title and abstract or the full text when necessary. Data were also independently extracted by the investigators. Any discrepancy was arbitrated by the senior investigator. Additionally, the risk of bias for each included RCT was assessed by the Cochrane Risk of Bias tool (Revman 5.2; http://methods.cochrane.org/bias/risk-bias-tool). The following data were collected from the studies: Name of the first author, publication year, country, number of patients, condition, therapeutic drugs, treatment dosage, and the HRs and confidence intervals (CIs) associated with the PFS and OS. Subsequently, the data regarding PFS and OS at 3, 6, 12, 18, 24, 30 and 36 months were collected from Kaplan-Meier curves using GetData 2.26 (https://getdata.sourceforge.net/download.).

### Research endpoint

Due to the incompleteness of data for OS, the primary endpoint was PFS rate at each time node generated by the Bayesian NMA.

### Data analysis

For the PFS and OS rates at each time node, odds ratios (ORs) were generated by NMA using STATA 17.0 MP (https://www.stata.com/statamp/), to make pairwise comparisons among regimens. Statistical significance was determined if the lower limit of the 95% CI was >1 or the upper limit was <1. Similarly, for the absolute PFS and OS values, the standard mean difference (SMD) was generated. Surface under the SUCRA was also formulated, where a higher SUCRA indicated a higher probability of being the superior treatment. However, whether the effect size between any pair with the corresponding SUCRAs reached the level of significance was determined by net-league table, also termed a matrix in algebra. Additionally, inconsistency and consistency tests were conducted to examine the existence of any inconsistency. Publication bias was also assessed by funnel plot.

For HRs of OS and PFS in each study, Napierian Logarithm HR (lnHR) and standard error of lnHR (selnHR) were calculated using STATA 17.0 MP. Subsequently these data (lnHR and selnHR for OS and PFS) were input into Rstudio 4.2.2 (https://cran.rstudio.com/bin/windows/base) using the ‘gemtc’ package to conduct a Bayesian NMA to generate pairwise HRs, SUCRA and matrix. Markov-chain Monte Carlo (MCMC) was used to obtain posterior distributions, with 2,000 burn-ins and 100,000 iterations of 4 for each chain and a thinning interval of 10 for each outcome. Brooks-Gelman-Rubin diagnostics and Trace and density plots were used to evaluate and visualize the convergence of the model over iterations. The random-effect model was used when conducting the NMA. However, to ensure the reliability of the data and minimize the impact of heterogeneity on the final results, a fixed-effects model was used when comparing direct and indirect comparison results (14).

## Results

### Characteristics of the included studies

**Figure 1.**
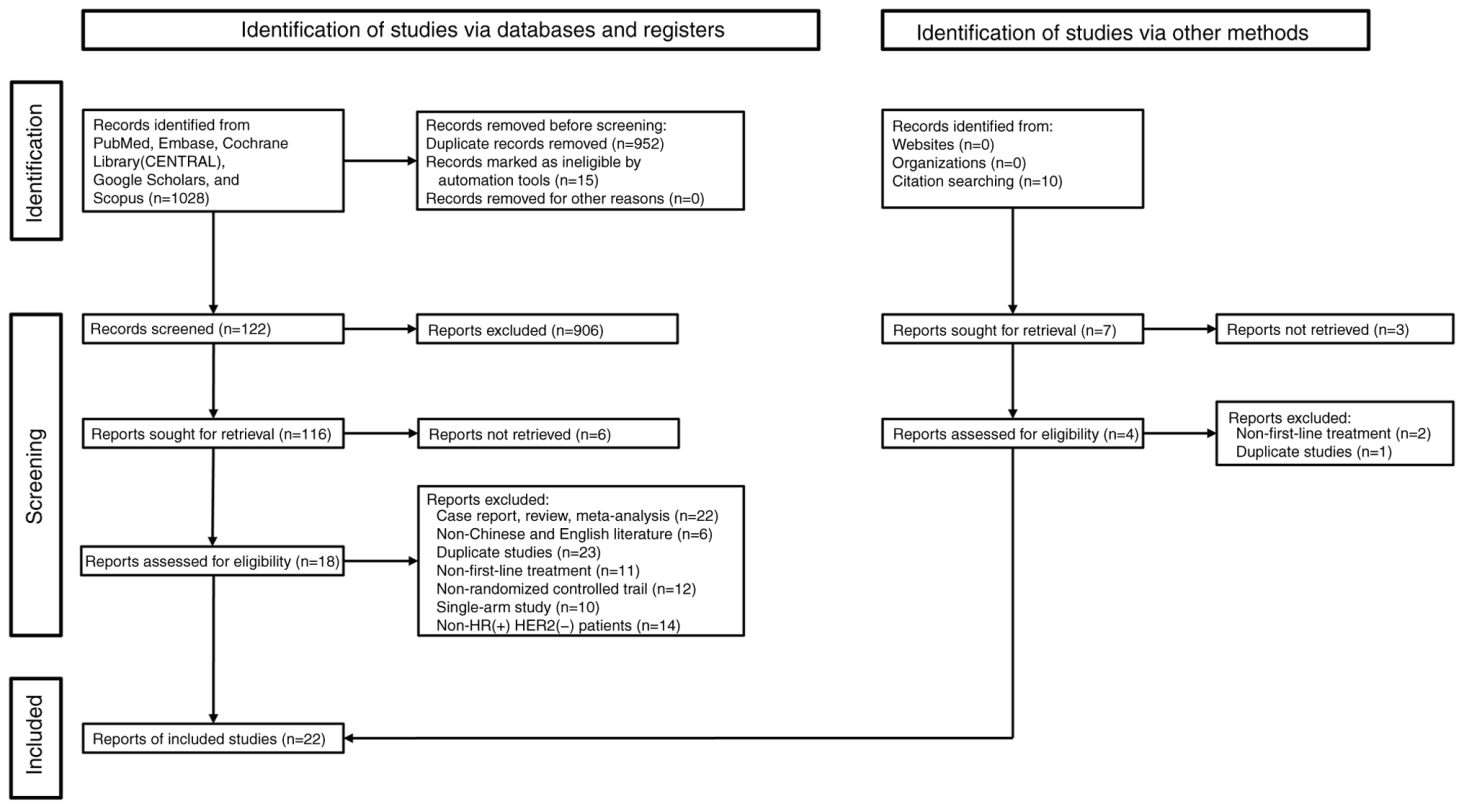
Preferred reporting items for systematic reviews and meta-analyses flowchart illustrating the selection of studies included in the present study. CENTRAL, cochrane central register of controlled trials; HR(+), hormone receptor-positive; HER2(−), receptor tyrosine-protein kinase erbB-2-negative.

A detailed description of the included studies can be found in Table I. Initially, 1,028 articles from all five databases were retrieved after meticulous screening; however, only 16 RCTs reporting 7,174 patients were eligible for the present study (reporting 1,292 patients with only bone metastasis, 3,294 patients with visceral metastasis, 2,416 patients with non-visceral metastasis, 2,512 patients with 1 or 2 metastasis sites, 1,596 patients with >2 metastasis sites and 1,109 patients were *de novo*; some patients belonged to several categories) (Fig. 1) (7,15–35). At total of 11 different maintenance regimens were evaluated within the included studies, namely, AI alone, abemaciclib plus AI (AbeAI), abemaciclib plus fulvestrant (AbeFul), dalpiciclib plus AI (DalpAI), fulvestrant (Ful), fulvestrant plus AI, lapatinib plus AI, palbociclib plus AI (PalboAI), palbociclib plus fulvestrant (PalboFul), ribociclib plus AI (RiboAI) and ribociclib plus fulvestrant (RiboFul). The included patients were from Asia, Europe, South/North America and Africa. As shown in Fig. S1, the risk of bias assessment results showed that there was no high risk of bias in the included studies.

### Primary analysis of PFS

#### PFS at each time node

**Figure 2.**
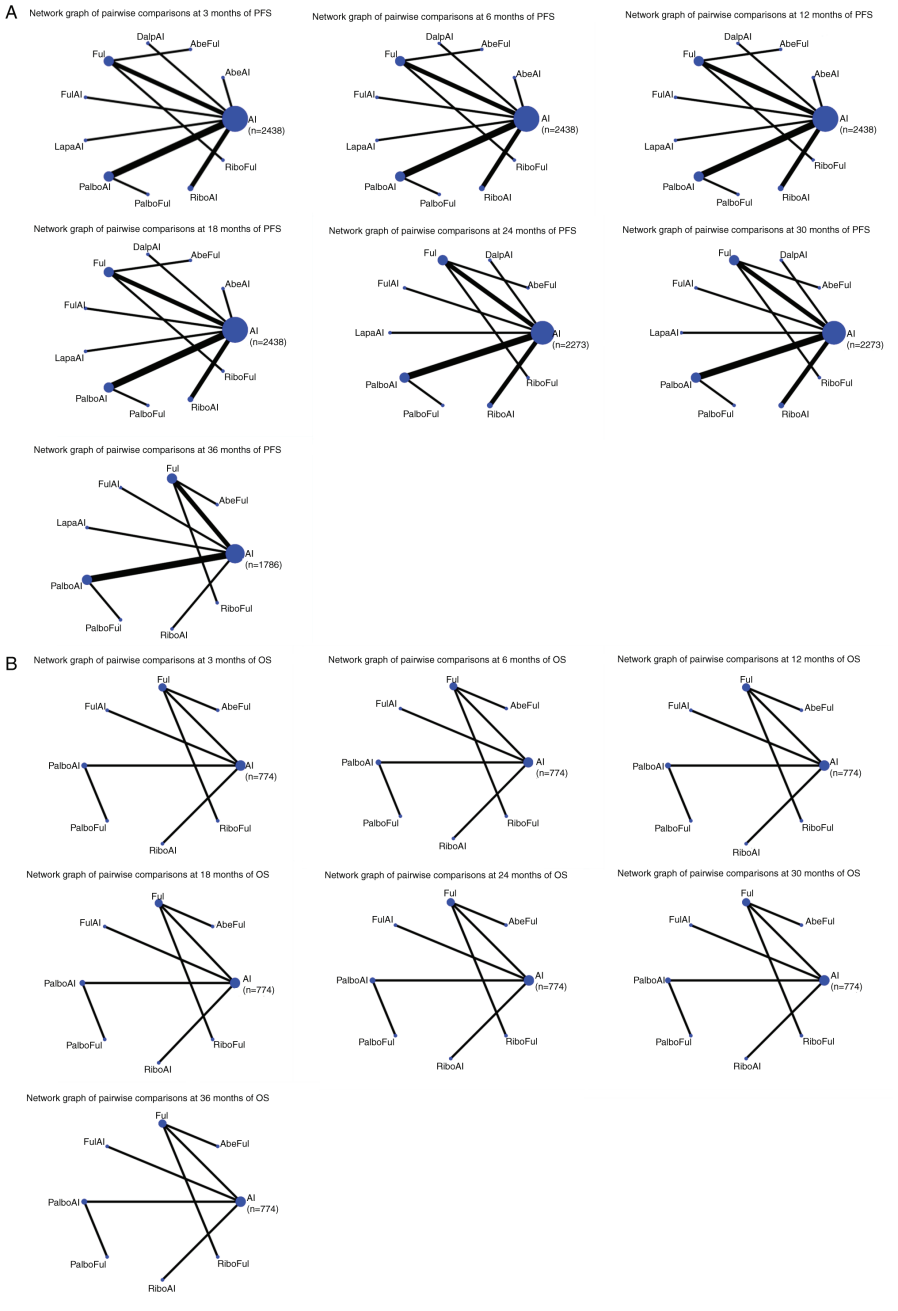
(A) Network graphs of the pairwise comparisons of regimens at each time point of the PFS curve. (B) Network graphs of the pairwise comparison of regimens at each time point of the OS curve. PFS, progression free survival; OS, overall survival; AI, aromatase inhibitor; AbeAI, abemaciclib plus AI; AbeFul, abemaciclib plus fulvestrant; DalpAI, dalpiciclib plus AI; Ful, fulvestrant; FulAI, fulvestrant plus AI; LapaAI, lapatinib plus AI; PalboAI, palbociclib plus AI; PalboFul, palbociclib plus fulvestrant; RiboAI, ribociclib plus AI; RiboFul, ribociclib plus fulvestrant.

Fig. 2A shows the network graphs from the pairwise comparison of regimens at each PFS time point. On the 3rd month, compared with AI, AbeAI (OR=4.92; 95% CI, 1.28–18.90) and PalboAI (OR=2.22; 95% CI, 1.10–4.47) significantly increased the 3-month PFS rate. Compared with the top SUCRA-ranked intervention AbeAI, PalboAI did not exhibit a significant advantage (OR=0.45; 95% CI, 0.10–2.05) (Table SI).

On the 6th month, compared with AI, only PalboAI (OR=2.39; 95% CI, 1.21–4.69), significantly increased the 6-month PFS rate (Table SII).

On the 12th month, compared with AI, the treatments performed as follows: AbeAI (OR=2.02; 95% CI, 1.11–3.70), RiboFul (OR=3.07; 95% CI, 1.22–7.71), PalboAI (OR=1.94; 95% CI, 1.34–2.79), RiboAI (OR=1.93; 95% CI, 1.21–3.07), AbeFul (OR=2.61; 95% CI, 1.25–5.44) and DalpAI (OR=2.07; 95% CI, 1.13–3.79). AbeAI, PalboAI, RiboAI, AbeFul and DalpAI did not exhibit a significant advantage when compared with the top SUCRA-ranked intervention RiboFul (Table SIII).

On the 18th month, compared with AI, the treatments performed as follows: AbeAI (OR=2.10; 95% CI, 1.18–3.73), RiboFul (OR=2.66; 95% CI, 1.19–5.96), PalboAI (OR=2.38; 95% CI, 1.65–3.44), RiboAI (OR=1.85; 95% CI, 1.20–2.85), AbeFul (OR=2.64; 95% CI, 1.25–5.59), DalpAI (OR=2.11; 95% CI, 1.18–3.76) and PalboFul (OR=2.13; 95% CI, 1.08–4.18). AbeAI, RiboFul, PalboAI, RiboAI, DalpAI and PalboFul did not show a significant advantage when compared with the top SUCRA-ranked intervention AbeFul (Table SIV).

On the 24th month, compared with AI, the treatments performed as follows: RiboFul (OR=2.12; 95% CI, 1.03–4.38), PalboAI (OR=2.39; 95% CI, 1.67–3.43), RiboAI (OR=1.84; 95% CI, 1.27–2.68), AbeFul (OR=4.01; 95% CI, 1.93–8.31), DalpAI (OR=2.36; 95% CI, 1.39–4.03), PalboFul (OR=2.16; 95% CI, 1.16–4.03) and Ful (OR=1.68; 95% CI, 1.11–2.54). RiboFul, PalboAI, RiboAI, DalpAI, PalboFul and Ful did not exhibit a significant advantage when compared with the top SUCRA-ranked intervention AbeFul (Table SV).

On the 30th month, compared with AI, the treatments performed as follows: RiboFul (OR=2.35; 95% CI, 1.34–4.10), PalboAI (OR=2.10; 95% CI, 1.62–2.74), RiboAI (OR=1.60; 95% CI, 1.24–2.06), AbeFul (OR=4.94; 95% CI, 2.62–9.33), DalpAI (OR=3.97; 95% CI, 2.59–6.08), PalboFul (OR=1.78; 95% CI, 1.15–2.78) and Ful (OR=1.62; 95% CI, 1.15–2.27). RiboFul, PalboAI, RiboAI, DalpAI, PalboFul and Ful did not exhibit a significant advantage when compared with the top SUCRA-ranked intervention AbeFul (Table SVI).

On the 36th month, compared with AI, the treatments performed as follows: PalboAI (OR=2.66; 95% CI, 1.37–5.18) and AbeFul (OR=6.21; 95% CI, 1.71–22.57). Compared with the top SUCRA-ranked intervention AbeFul, PalboAI did not exhibit a significant advantage (OR=0.43; 95% CI, 0.10–1.82) (Table SVII).

As such, the regimen with the most significant effect on PFS between 3–36 months compared with AI was PalboAI (Table II).

### HR of PFS

**Figure 3.**
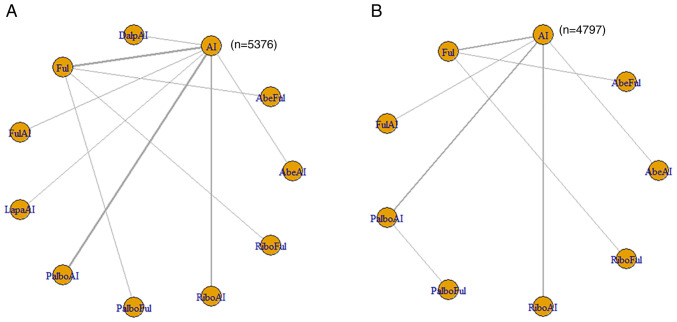
(A) Network meta-analysis plots for HR of progression free survival. (B) Network meta-analysis plots for HR of overall survival. HR, hazard ratio; AI, aromatase inhibitor; AbeAI, abemaciclib plus AI; AbeFul, abemaciclib plus fulvestrant; DalpAI, dalpiciclib plus AI; Ful, fulvestrant; FulAI, fulvestrant plus AI; LapaAI, lapatinib plus AI; PalboAI, palbociclib plus AI; PalboFul, palbociclib plus fulvestrant; RiboAI, ribociclib plus AI; RiboFul, ribociclib plus fulvestrant.

A total of 16/22 articles reported outcomes associated with the HRs of PFS. The 11 included interventions were compared directly and indirectly. The corresponding network graph is shown in Fig. 3A, and detailed results are shown in Table SVIII. The interventions that exhibited significant differences compared with AI were AbeAI [HR=1.96; 95% credible interval (Crl), 1.25–3.09], AbeFul (HR=2.45; 95% Crl, 1.59–3.93), DalpAI (HR=1.96; 95% Crl, 1.29–2.98), Ful (HR=1.4; 95% Crl, 1.11–1.82), PalboAI (HR=1.7; 95% Crl, 1.36–2.16), PalboFul (HR=2.93; 95% Crl, 1.68–5.21), RiboAI (HR=1.76; 95% Crl, 1.35–2.3) and RiboFul (HR=2.23; 95% Crl, 1.4–3.65). Compared with the top SUCRA-ranked intervention, PalboFul, 7 treatments (PalboFul, AbeFul, DalpAI, PalboAI, PalboFul, RiboAI and RiboFul) were included in the first echelon; fulvestrant was included in the second echelon.

For the HR of PFS, the Brooks-Gelman Rubin convergence diagnostic revealed that the inferential iterations for each MCMC were stable and reproducible. The history feature was also used to confirm the convergence of the model in all outcomes. Detailed results are presented in Figs. S2 and S3.

### Absolute PFS value

**Figure 4.**
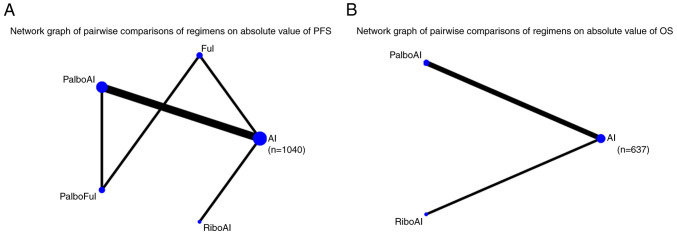
(A) Network meta-analysis plot for the absolute PFS value. (B) Network meta-analysis plot for the absolute OS value. PFS, progression free survival; OS, overall survival; AI, aromatase inhibitor; Ful, fulvestrant; PalboAI, palbociclib plus AI; PalboFul, palbociclib plus fulvestrant; RiboAI, ribociclib plus AI.

In total, 7 articles reported outcomes on the absolute PFS value. The SMD was used to compare the 5 eligible studies directly and indirectly (the treatments reported in 2 articles failed to connect in the network comparison). The network graph is shown in Fig. 4A and detailed results are shown in Table SIX. The interventions that exhibited significant differences compared with AI were PalboAI (SMD=0.37; 95% Crl, 0.25–0.49) and RiboAI (SMD=0.62; 95% Crl, 0.28–0.96).

### Primary analysis of OS

#### OS at each time node

Compared with AI, only RiboFul (OR=2.66; 95% Cl, 1.37–5.18) exhibited a significant advantage at 18 months. The network graphs of pairwise comparisons among regimens on each time point of the OS curve are shown in Fig. 2B, and detailed results are listed in Table SX, Table SXI, Table SXII, Table SXIII, Table SXIV, Table SXV, Table SXVI.

### HR of OS

Of the 22 articles, 12 reported outcomes related to the HR of OS. The 11 included interventions were compared directly and indirectly. The only intervention that exhibited a significant difference compared with AI was RiboFul (HR=1.99; 95% Crl, 1.14–3.47; Table SXVII). The network graph is shown in Fig. 3B, and detailed results are listed in Table SV. For the HR of OS, the Brooks-Gelman Rubin convergence diagnostic revealed that the inferential iterations for each MCMC were stable and reproducible. The history feature was also used to confirm the convergence of the model in all outcomes. Detailed results are presented in Figs. S4 and S5.

### Absolute value of OS

Only 3 articles reported outcomes related to the absolute OS value. The SMD was used to compare the three included interventions directly and indirectly. The intervention measure with a significant difference compared with AI was RiboAI (SMD=0.17; 95% Crl, 0.02–0.32). The network graph is shown in Fig. 4B and detailed results are shown in Fig. S6.

### Inconsistency tests, heterogeneity analysis and small sample effect tests

No inconsistency and heterogeneity were observed between studies included in the present Bayesian NMA. The small sample effect was explored by a network funnel plot, and the small sample effect was not observed. P<0.05 was considered to indicate a statistically significant difference (Fig. S7, Fig. S8, Fig. S9).

## Discussion

### Current status of breast cancer treatment

Due to the heterogeneity of tumor cells, different differentiation cycles have distinct characteristics. Different antitumor drugs target different cell cycles, and thus the comprehensive treatment of tumors is complicated (36). For example, certain drugs act on nucleic acid replication and certain endocrine drugs act on different targets. During the differentiation process of tumor cells, resistance to a certain pharmacological mechanism may occur, which means a single antitumor treatment is ineffective (37).

In recent years, several new surgical and medicinal technologies and drugs have emerged for the treatment of BC, which have brought great benefits to patients. Even small tumors that are non-palpable can be accurately removed through ultrasound combined with marker positioning technology to reduce damage while ensuring the margins (38,39). Patients with axillary lymph node-positive tumors typically require further dissection, and the most common complication is seroma, which makes patients feel uncomfortable and can cause further infection. A new hemostatic device (Thunderbeat) can effectively reduce the occurrence of serum swelling (40). There are also several treatments for mBC. The aim of the present study was to discuss the best options for patients with ER^+^/HER2^−^ mBC.

To the best of our knowledge, the present study reports the first Bayesian NMA comparing relative efficacy of all current available maintenance therapies for HR^+^/HER2^−^ mBC. The findings were as follows:

### Core findings

#### Transverse comparisons

Compared with AI, the interventions that exhibited significantly different effects on PFS were PalboAI and AbeAI on the 3rd month of follow-up. Similarly, PalboAI and AbeFul exhibited significantly different effects on the 36th month, and only PalboAI exhibited significantly different effects on the 6th month. Riboful, PalboAI, RiboAI, AbeAI and DalpAI exhibited significantly different effects between months 12 and 30. PalboAI demonstrated benefits at each time node between months 3 and 36. Based on the Bayesian NMA of HRs and the absolute value time as a covariate of PFS, PalboAI also exhibited benefits.

#### Longitudinal comparisons

With regards to the PFS up to 36 months of follow-up, and compared with AI therapy, AbeAI exhibited a similar efficacy to that of PalboAI on the 3rd month. DalpAI also had improved results compared with AI, but due to the lack of data, it was not possible to analyze whether the significance persisted until the 36th month. AbeFul had improved results compared with AI between the 12th and 36th months, but it did not demonstrate a significant advantage between the 3rd and 6th months. Similarly, RiboAI and RiboFul revealed a significant advantage between the 12th and 30th months. PalboAI was the only regimen that demonstrated significant efficacy between the 3rd and 36th months of follow-up.

Regarding OS up to the 36th month of follow-up, it was found that most of the studies did not report this endpoint. Due to the lack of data, the OS results were not considered in the present study.

#### Clinicopathological correlation

CDK4/6 inhibitors efficiently and accurately inhibit the activity of CDK4/6 kinases in BC cells to block the phosphorylation of retinoblastoma protein, thus blocking the progression of the cell cycle from the G1 to the S phase, in turn inhibiting the proliferation of tumor cells. CDK4/6 inhibitors also inhibit the expression of the upstream estrogen receptor signaling pathway, and there is a synergistic effect of CDK4/6 inhibitors combined with endocrine therapy to delay and reverse endocrine drug resistance (41). The different CDK4/6 inhibitors have comparable molecular weights and share the same core group. The substituent of pabociclib is relatively large and shows high kinase selectivity (42). Pabociclib has a significant pharmacokinetic profile [volume of distribution of 2583 L (ribociclib 1090 L, abemaciclib 690 L), bioavailability of 46% (ribociclib not reported, abemaciclib 45%) protein binding ratio of 85% (ribociclib 70%, abemaciclib 93%) and half-life of 29±5 h (ribociclib 30–55 h, abemaciclib 18.3 h] (43). These properties may explain the early benefits and sustained effects at each point of the PFS time nodes observed in this analysis.

#### Feasibility analysis

A common adverse effect of CDK4/6 inhibitors is neutropenia. In contrast to the mechanism of chemotherapy-induced myelosuppression, CDK4/6 inhibitors do not cause neutrophil precursor death, reducing the risk of febrile neutropenia (44). However, close clinical monitoring and management is recommended. The reported incidence of hematological adverse events associated with CDK4/6 inhibitors is low and the severity is milder: i) Neutropenia, palbociclib 79.5%, abemaciclib 80% and dalpiciclib 99%; ii) leucopenia, palbociclib 39%, abemaciclib 76.1% and dalpiciclib 98.3%; iii) anemia, palbociclib 24.1%, abemaciclib 62% and dalpiciclib 66.9%; and iv) thrombocytopenia, palbociclib 15.5%, abemaciclib 44.4% and dalpiciclib 53.3% (16,45,46).

Diarrhea is the most common gastrointestinal adverse event associated with all available CDK4/6 inhibitors, with the highest incidence observed in patients treated with abemaciclib (80%); therefore, in patients with poor gastrointestinal function, it is recommended to prioritize other CDK4/6 inhibitors with less probability of this adverse event, such as ribociclib (20%), palbociclib (10.7%) or dalpiciclib (10.4%) (16,45–47). There are no reports on the metabolic safety of pabociclib in diabetic patients. However, it has been reported that abemaciclib causes severe hypoglycemia and ribociclib causes lactic acidosis (48–50).

Of all the available CDK4/6 inhibitors, palbociclib has a relatively low incidence of overall adverse events and, since all palbociclib combination regimens are oral formulations, this therapy is medically accessible and suitable for all patient groups. In particular, all palbociclib regimens have been included in China's national health insurance catalogue, which reduces the financial burden for patients.

#### Limitations

First, the sample sizes of certain included studies were inadequate, resulting in the small sample effect and potential bias. Second, in the analyses of absolute OS/PFS values, certain studies had not reported mean/median OS/PFS and 95% confidence intervals or the interquartile range. These studies had to be excluded, which potentially shrank the sample size by another means, eventually increasing random error. Third, the limited resolution of survival curve images in certain studies was compromised. Finally, the quality of some of the studies was low, bringing potential interference.

#### Perspectives

We hope that the design of future clinical trials will be more precise and the final OS data will be reported. Adverse events should be evaluated after long-term follow-ups and could be compared at each time node. When making clinical decisions, adverse effects should be considered. FDA Adverse Event Reporting System database can provide some information on these adverse events.

In the present study, significant heterogeneity was not observed when analyzing the heterogeneity of the computational model, indicating that the baseline differences among patients included in the NMA were not sufficient to affect the final results. The patients included in the present study all had metastatic disease and accepted first-line treatments; therefore, the results are applicable to these first-line patients with metastases. In conclusion, considering the benefit of treatment on PFS emerged earlier and over a long period of time, PalboAI should be recommended as the optimal therapy in HR^+^/HER2^−^ mBC. However, it is necessary to design more RCTs to confirm this result.

## Supplementary Material

Supporting Data

Supporting Data

## Figures and Tables

**Table I.** Characteristics of first-line systemic therapy for hormone receptor-positive and receptor tyrosine-protein kinase erbB-2-negative metastatic breast cancer studies included in the Bayesian network meta-analysis.

Trial acronym	First author, year	Country	Treatment	Sample size	Dosage	Outcomes	(Refs.)
-	Wang *et al*, 2021	China	Fulvestrant/AI	77/67	Fulvestrant 500 mg on days 0, 14 and 28 and every 28±3 days thereafter/Exemestane 25 mg daily.	PFS	(15)
DAWNA-2	Zhang *et al*, 2023	China	Dalpiciclib + AI/AI	303/153	Dalpiciclib (150 mg daily for 3 weeks, followed by 1 week off + Letrozole 2.5 mg or Anastrozole 1 mg daily/Letrozole 2.5 mg or Anastrozole 1 mg daily.	PFS	(16)
FALCON	Robertson *et al*, 2016	UK	Fulvestrant/AI	230/232	Fulvestrant 500 mg on days 0, 14 and 28 and every 28 days thereafter/Anastrozole 1 mg daily.	PFS, OS	(17)
FIRST	Ellis *et al*, 2015	USA	Fulvestrant/AI	102/103	Fulvestrant 500 mg on days 0, 14 and 28 and every	OS	(7)
	Robertson *et al*, 2012	UK			28 days thereafter/Anastrozole 1 mg daily.	PFS	(18)
-	Llombart *et al*, 2021	Spain	Fulvestrant + Palbociclib/AI + Palbociclib	243/243	Palbociclib 125 mg daily (in cycles of 3 weeks of treatment followed by 1 week off) + Fulvestrant 500 mg on days 1, 15, 29 and once monthly thereafter/Palbociclib 125 mg daily (in cycles of 3 weeks of treatment followed by 1 week off) + Letrozole 2.5 mg daily.	PFS, OS	(19)
-	Johnston *et al*, 2009	UK	Lapatinib + AI/AI	478/474	Letrozole 2.5 mg + Lapatinib 1,500 mg daily/Letrozole 2.5 mg daily.	PFS, OS	(20)
MONARCH-3	Goetz *et al*, 2022	USA	Abemaciclib +	328/165	Abemaciclib 150 mg twice daily continuous	OS	(21)
	Goetz *et al*, 2017	USA	AI/AI		schedule + Anastrozole 1 mg or Letrozole 2.5 mg daily/Anastrozole 1 mg or Letrozole 2.5 mg daily.	PFS	(22)
FLIPPER	Albanell *et al*, 2021	Spain	Palbociclib + Fulvestrant/Fulvestrant	94/95	Palbociclib 125 mg daily (in cycles of 3 weeks of treatment followed by 1 week off) + Fulvestrant 500 mg on days 1, 15, 29 and once monthly thereafter/Fulvestrant 500 mg on days 1, 15, 29 and once monthly thereafter.	PFS	(28)
MONARCH-2	Neven *et al*, 2021	Belgium	Abemaciclib + Fulvestrant/Fulvestrant	265/133	Abemaciclib 150 mg twice daily + Fulvestrant 500 mg on days 0, 14 and 28 and every 28 days thereafter/Fulvestrant 500 mg on days 0, 14 and 28 and every 28 days thereafter.	PFS, OS	(23)
PALOMA-1	Finn *et al*, 2015	USA	Palbociclib + AI/AI	84/81	Palbociclib 125 mg daily (3/1 schedule) + Letrozole	PFS	(24)
	Finn *et al*, 2020	USA			2.5 mg daily, continuous/Letrozole 2.5 mg daily, continuous.	OS	(25)
MONALEESA-3	Slamon *et al*, 2021	USA	Ribociclib + Fulvestrant/Fulvestrant	237/128	Ribociclib 600 mg (once daily for 21 days, followed by 7 days off, 28 days for a cycle) + Fulvestrant 500 mg on days 0, 14 and 28 and every 28 days thereafter/Fulvestrant 500 mg on days 0, 14 and 28 and every 28 days thereafter.	PFS, OS	(30)
MONALEESA-2	Hortobagy *et al*, 2022	USA	Ribociclib + AI/AI	334/334	Ribociclib (600 mg, once daily for 21 days,	OS	(26)
	Hortobagyi *et al*, 2018	USA			followed by 7 days off, 28 days for a cycle) + Letrozole 2.5 mg daily/Letrozole 2.5 mg daily.	PFS	(31)
PALOMA-4	Xu *et al*, 2022	China	Palbociclib + AI/AI	168/171	Palbociclib 125 mg daily (3 weeks on, 1 week off) + Letrozole 2.5 mg daily, continuously/Letrozole 2.5 mg daily, continuously.	PFS	(27)
MONALEESA-7	Tripathy *et al*, 2018	USA	Ribociclib + AI/AI	335/337	Ribociclib 600 mg (once daily for 21 days, followed	PFS	(29)
	Lu *et al*, 2021	China			by 7 days off, 28 days for a cycle) + Letrozole 2.5 mg or Anastrozole 1 mg, daily/Letrozole 2.5 mg or Anastrozole 1 mg, daily.	OS	(34)
PALOMA-2	Finn *et al*, 2022	USA	Palbociclib +	444/222	Palbociclib (125 mg/day; 3 weeks on, 1 week off) +	OS	(32)
	Rugo *et al*, 2019	USA	AI/AI		Letrozole 2.5 mg daily, continuously/Letrozole 2.5 mg daily, continuously.	PFS	(33)
FACT	Bergh *et al*, 2012	Sweden	Fulvestrant + AI/AI	258/256	Fulvestrant 500 mg on days 0, 14 and 28 and every 28 days thereafter/Anastrozole 1 mg orally daily.	PFS, OS	(35)

PFS, progression free survival; OS, overall survival; AI, aromatase inhibitor.

**Table II.** Progression free survival for interventions that were significant compared with AI.

Method	Control group	AbeAI, HR (CI)	RiboFul, HR (CI)	PalboAI, HR (CI)	RiboAI, HR (CI)	AbeFul, HR (CI)	DalpAI, HR (CI)	LapaAI, HR (CI)	PalboFul, HR (CI)	FulAI, HR (CI)	Ful, HR (CI)
3M	AI	4.92	x	2.22	x	x	x	x	x	x	x
		(1.28–18.90)		(1.10–4.47)							
6M	AI	x	x	2.39	x	x	x	x	x	x	x
				(1.21–4.69)							
12M	AI	2.02	3.07	1.94	1.93	2.61	2.07	x	x	x	x
		(1.11–3.70)	(1.22–7.71)	(1.34–2.79)	(1.21–3.07)	(1.25–5.44)	(1.13–3.79)				
18M	AI	2.10	2.66	2.38	1.85	2.64	2.11	x	2.13	x	x
		(1.18–3.73)	(1.19–5.96)	(1.65–3.44)	(1.20–2.85)	(1.25–5.59)	(1.18–3.76)		(1.08–4.18)		
24M	AI	NR	2.12	2.39	1.84	4.01	2.36	x	2.16	x	1.68
			(1.03–4.38)	(1.67–3.43)	(1.27–2.68)	(1.93–8.31)	(1.39–4.03)		(1.16–4.03)		(1.11–2.54)
30M	AI	NR	2.35	2.10	1.60	4.94	3.97	x	1.78	x	1.62
			(1.34–4.10)	(1.62–2.74)	(1.24–2.06)	(2.62–9.33)	(2.59–6.08)		(1.15–2.78)		(1.15–2.27)
36M	AI	NR	x	2.66	x	6.21	NR	x	x	x	x
				(1.37–5.18)		(1.71–22.57)					
HR	AI	1.96	2.23	1.7	1.76	2.45	1.96	x	2.93	x	1.4
		(1.25–3.09)	(1.4–3.65)	(1.36–2.16)	(1.35–2.3)	(1.59–3.93)	(1.29–2.98)		(1.68–5.21)		(1.11–1.82)
Absolute value	AI	NR	NR	0.37	0.62	NR	NR	NR	NR	NR	NR
				(0.25–0.49)	(0.28–0.96)						

M, month; AI, aromatase inhibitor; AbeAI, abemaciclib plus AI; AbeFul, abemaciclib plus fulvestrant; DalpAI, dalpiciclib plus AI; Ful, fulvestrant; FulAI, fulvestrant plus AI; LapaAI, lapatinib plus AI; PalboAI, palbociclib plus AI; PalboFul, palbociclib plus fulvestrant; RiboAI, ribociclib plus AI; RiboFul, ribociclib plus fulvestrant; HR, hazard ratio; CI, confidence interval; NR, not reported; x:, no significant advantage.

## Data Availability

The data generated in the present study may be requested from the corresponding author.
