# Supplementary material for: First‑line endocrine therapy for hormone receptor positive and HER‑2 negative metastatic breast cancer: A Bayesian network meta‑analysis
Source: Oncol Lett. 2024 Aug 28;28(5):513. doi: 10.3892/ol.2024.14646 (PMC11378012; doi:10.3892/ol.2024.14646)

Figure S1. (A) Summary of quality assessments using the Cochrane Risk of Bias Tool 2. (B) Risk of bias for the included studies

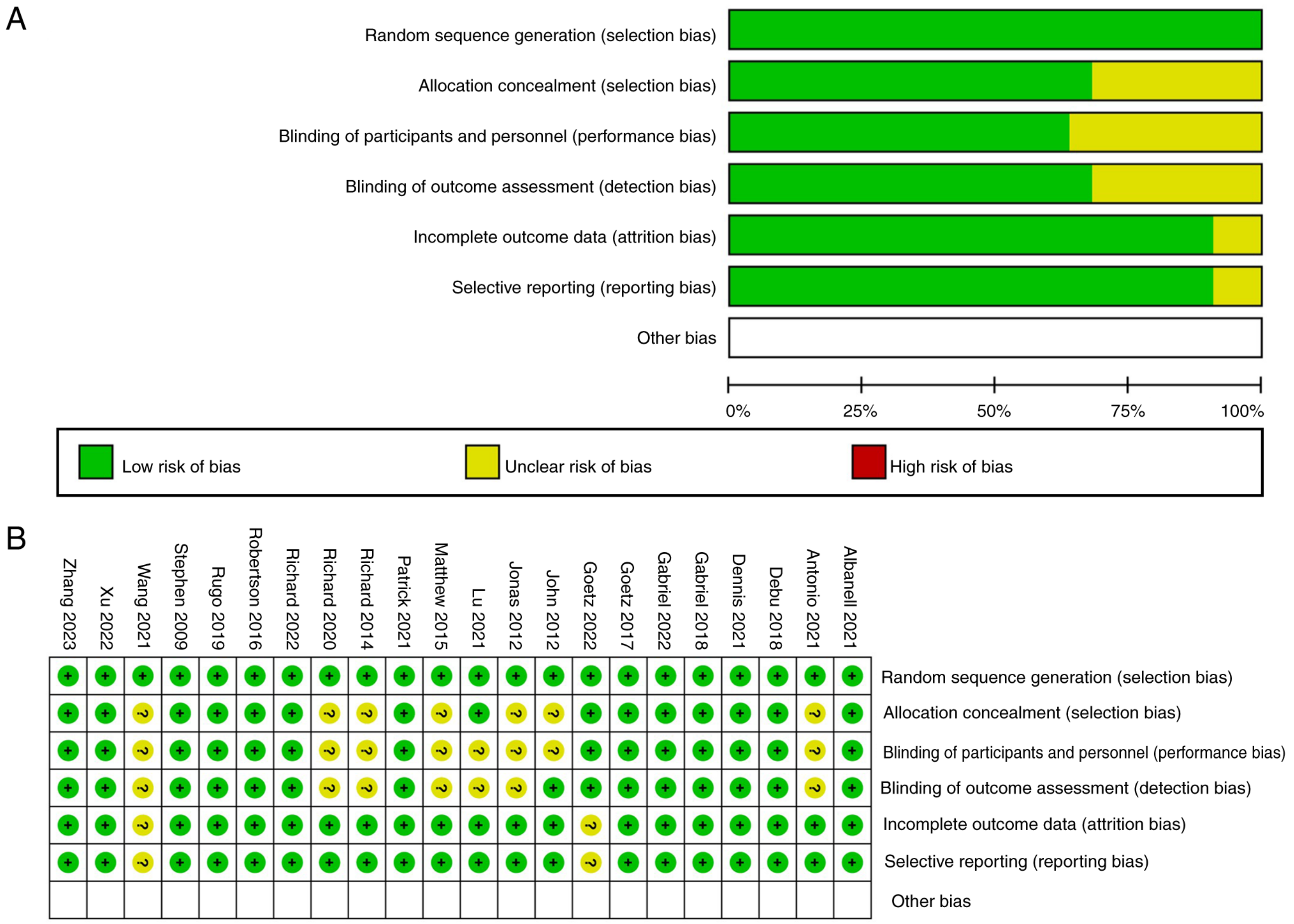

Figure S2. Convergence of the three Markov Chain Monte Carlo chains established by the history feature for progression free survival. AI, aromatase inhibitor; AbeAI, abemaciclib plus AI; AbeFul, abemaciclib plus fulvestrant; DalpAI, dalpiciclib plus AI; Ful, fulvestrant; FulAI, fulvestrant plus AI; LapaAI, lapatinib plus AI; PalboAI, palbociclib plus AI; PalboFul, palbociclib plus fulvestrant; RiboAI, ribociclib plus AI; RiboFul, ribociclib plus fulvestrant.

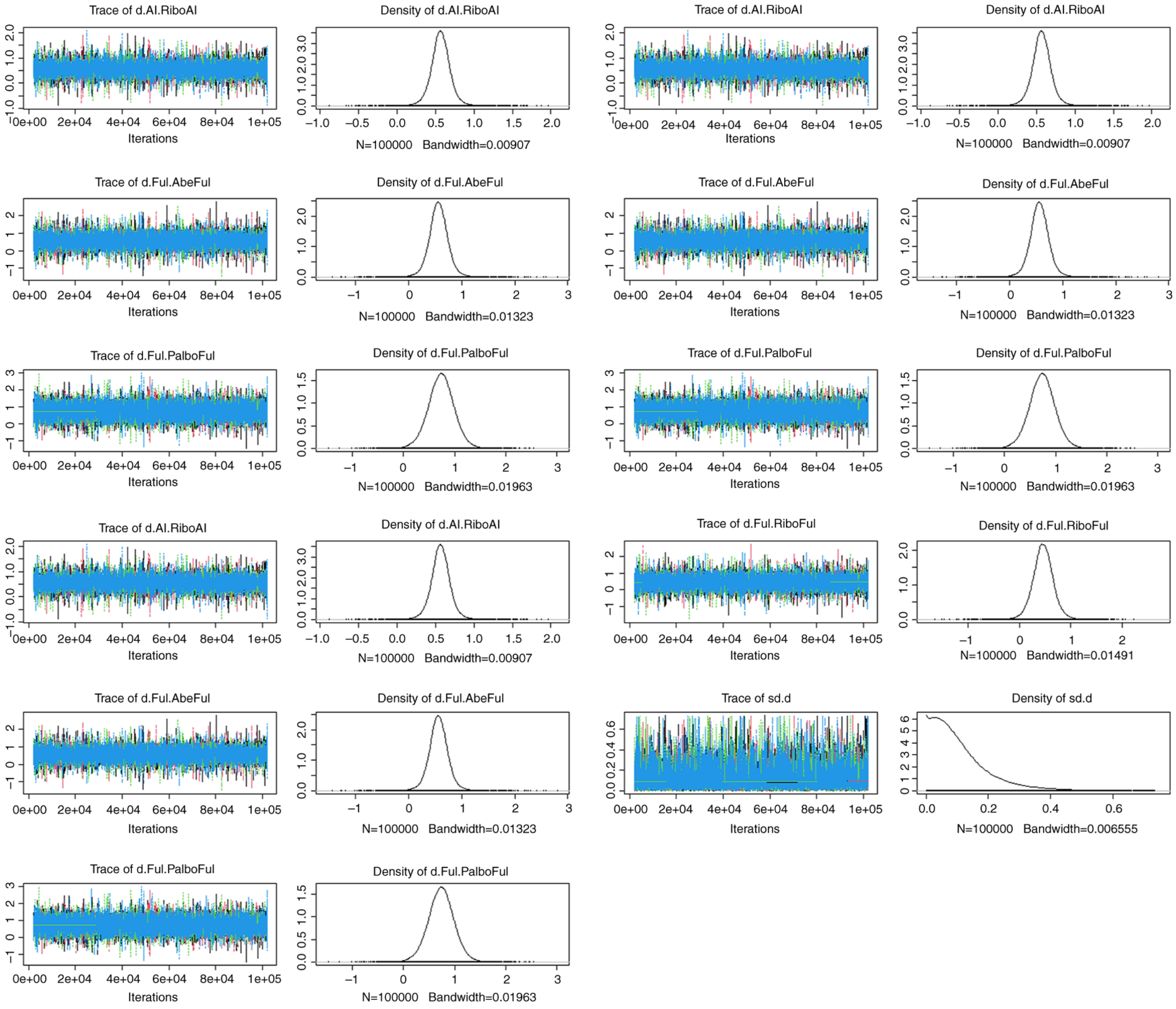

Figure S3. Convergence of the three Markov Chain Monte Carlo chains established by the Brooks-Gelman-Rubin diagnostic for progression free survival. AI, aromatase inhibitor; AbeAI, abemaciclib plus AI; AbeFul, abemaciclib plus fulvestrant; DalpAI, dalpiciclib plus AI; Ful, fulvestrant; FulAI, fulvestrant plus AI; LapaAI, lapatinib plus AI; PalboAI, palbociclib plus AI; PalboFul, palbociclib plus fulvestrant; RiboAI, ribociclib plus AI; RiboFul, ribociclib plus fulvestrant.

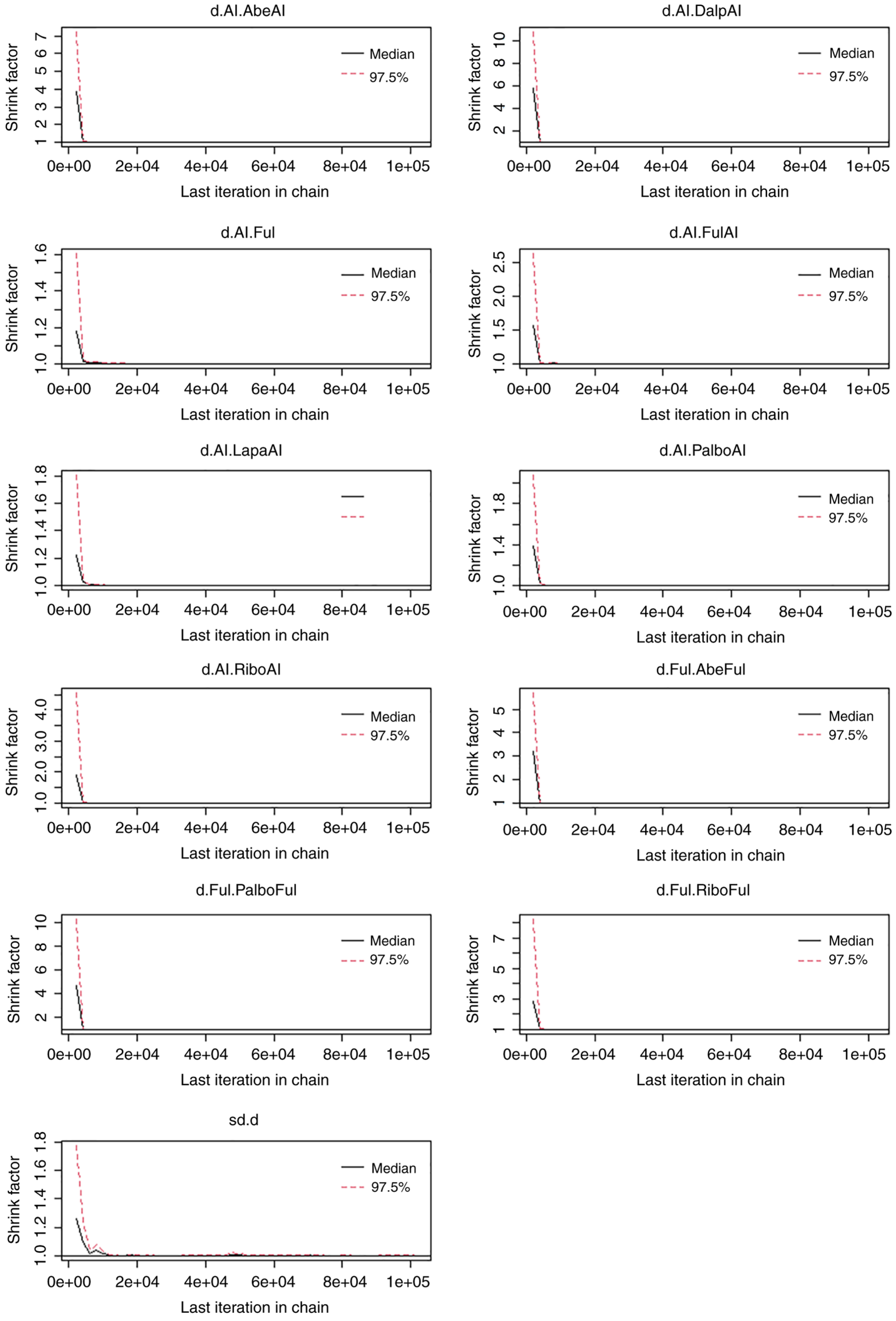

Figure S4. Convergence of the three Markov Chain Monte Carlo chains established by the history feature for overall survival. AI, aromatase inhibitor; AbeAI, abemaciclib plus AI; AbeFul, abemaciclib plus fulvestrant; DalpAI, dalpiciclib plus AI; Ful, fulvestrant; FulAI, fulvestrant plus AI; LapaAI, lapatinib plus AI; PalboAI, palbociclib plus AI; PalboFul, palbociclib plus fulvestrant; RiboAI, ribociclib plus AI; RiboFul, ribociclib plus fulvestrant.

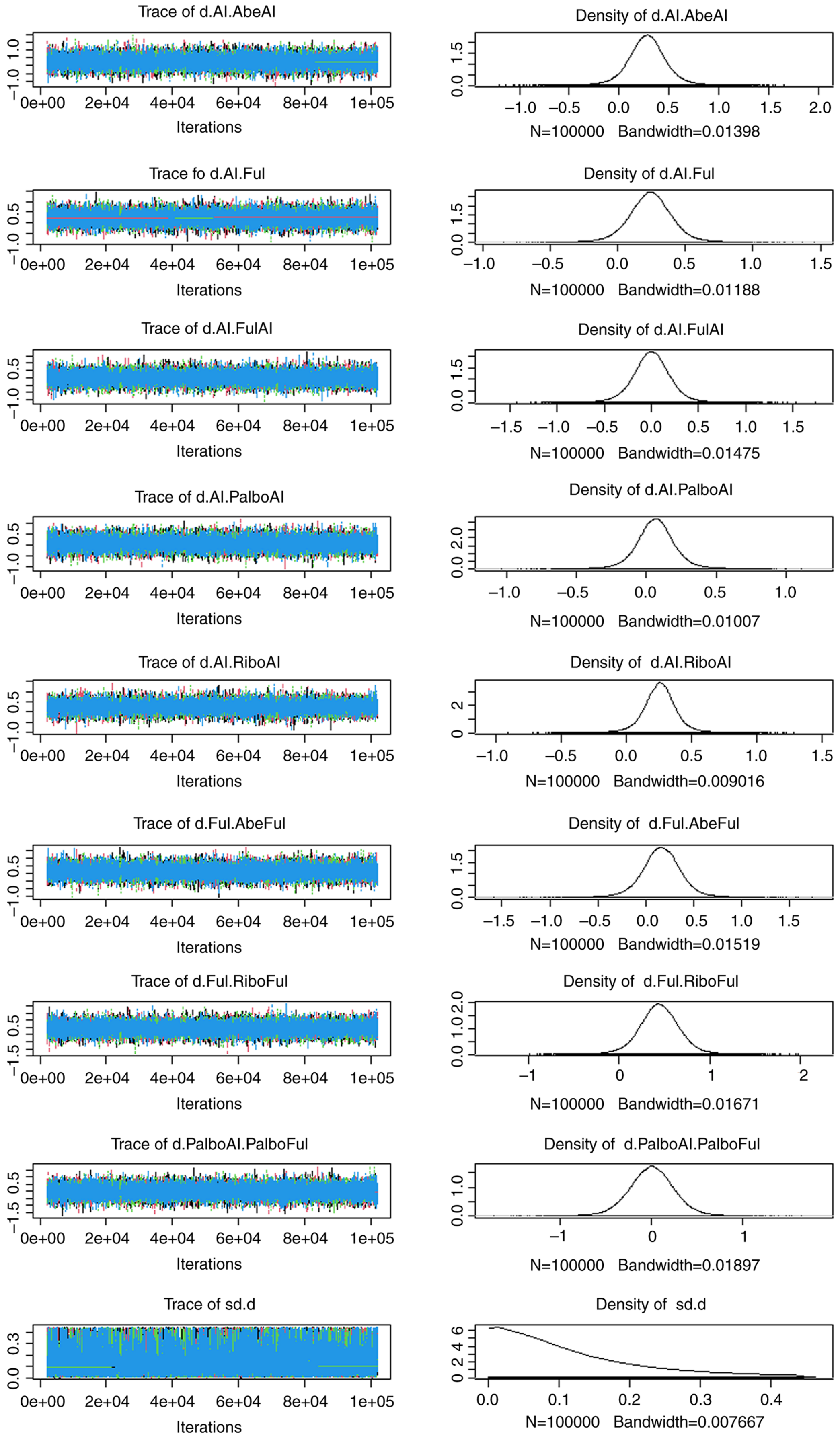

Figure S5. Convergence of the three Markov Chain Monte Carlo chains established by the Brooks-Gelman-Rubin diagnostic for overall survival. AI, aromatase inhibitor; AbeAI, abemaciclib plus AI; AbeFul, abemaciclib plus fulvestrant; DalpAI, dalpiciclib plus AI; Ful, fulvestrant; FulAI, fulvestrant plus AI; LapaAI, lapatinib plus AI; PalboAI, palbociclib plus AI; PalboFul, palbociclib plus fulvestrant; RiboAI, ribociclib plus AI; RiboFul, ribociclib plus fulvestrant.

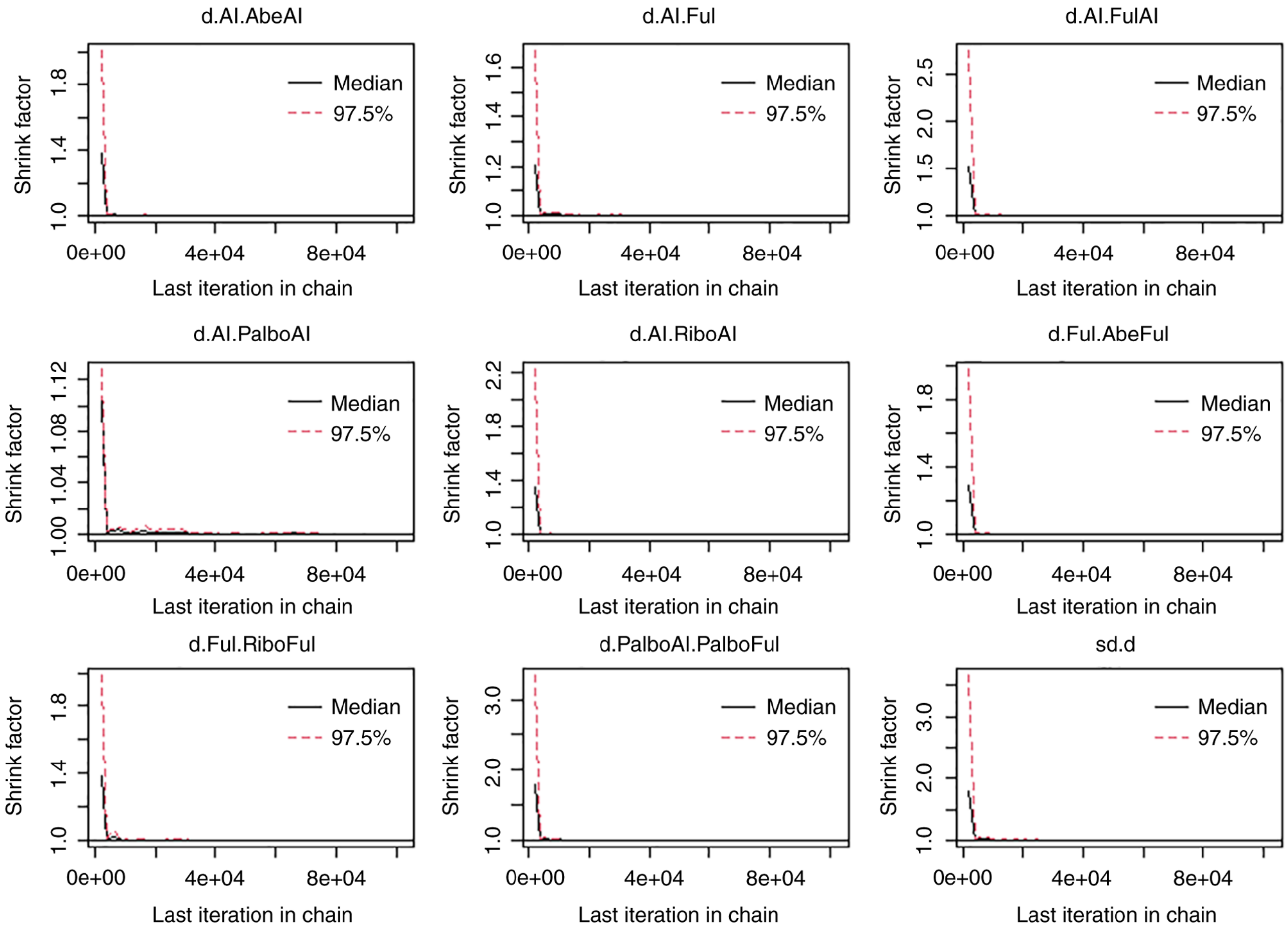

Figure S6. Network forest plot of the pairwise comparisons of regimens on the absolute OS value. OS, overall survival; CI, confidence interval; AI, aromatase inhibitor; PalboAI, palbociclib plus AI; RiboAI, ribociclib plus AI.

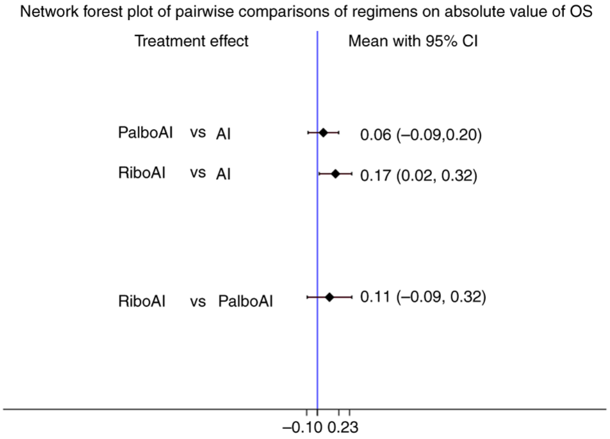

Figure S7. Network funnel plots of the pairwise comparisons at each time point of PFS. PFS, progression free survival.

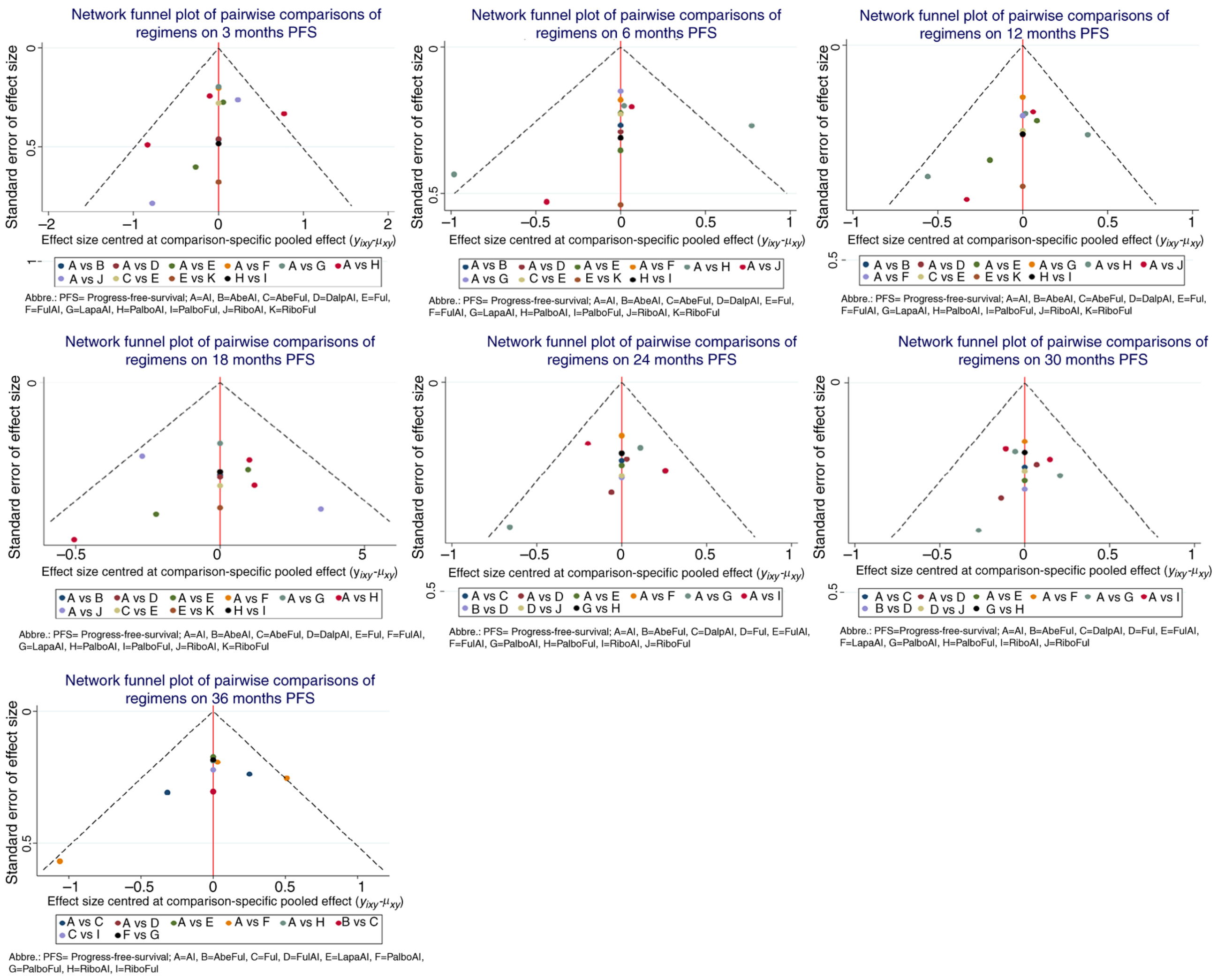

Figure S8. Network funnel plots of the pairwise comparisons at each time point of OS. OS, overall survival.

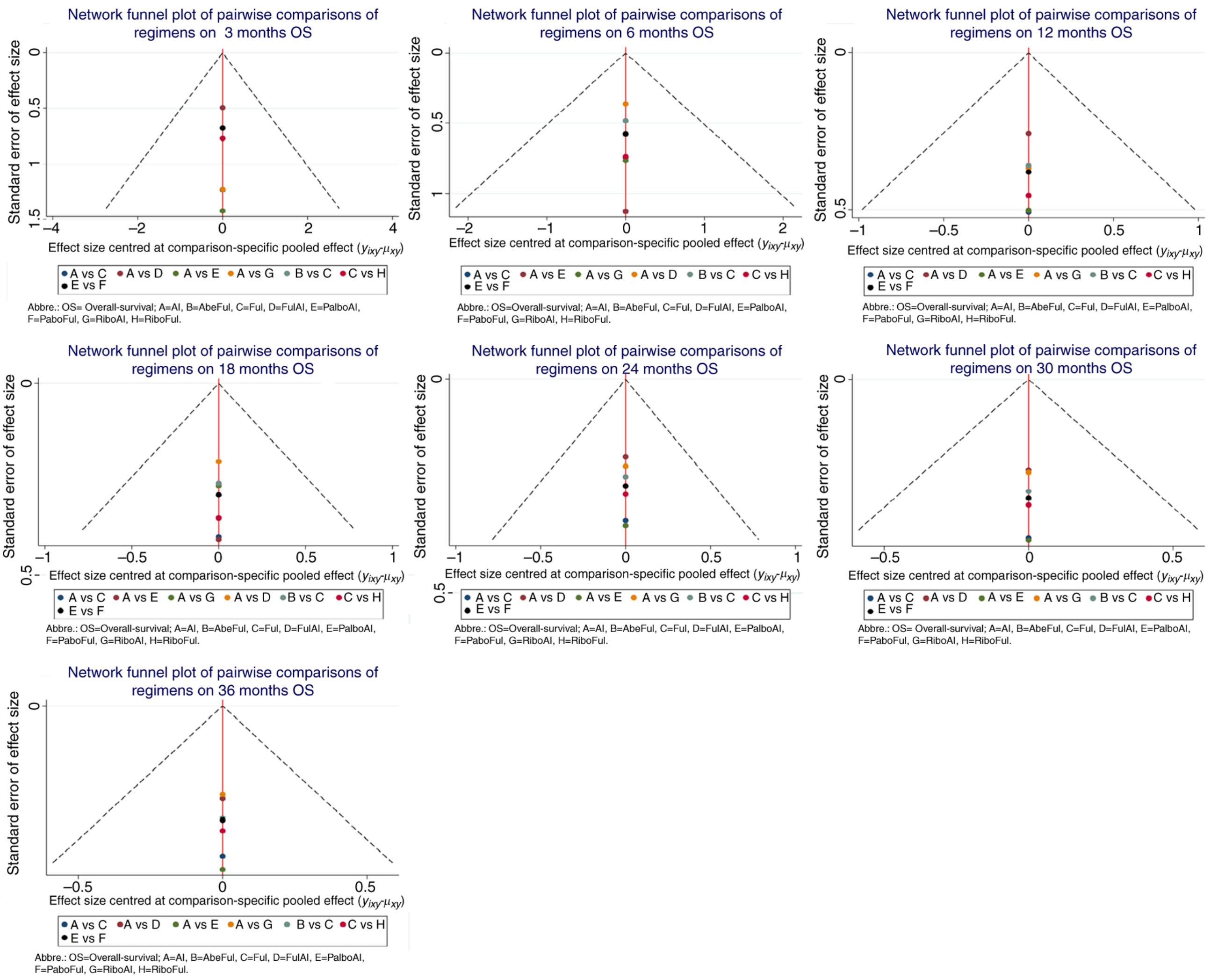

Figure S9. (A) Network funnel plot of the pairwise comparisons of regimens on the absolute PFS value. (B) Network funnel plot of the pairwise comparisons of regimens on absolute OS value. PFS, progression free survival; OS, overall survival.

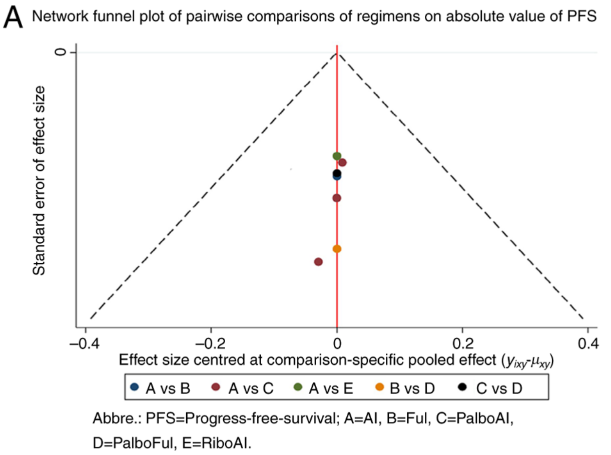

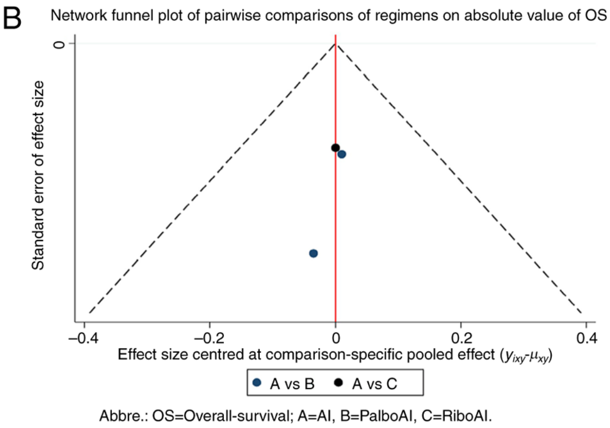

Supplement: Supporting Data [file Supplementary_Data1.pdf]
