# Supplementary material for: First‑line endocrine therapy for hormone receptor positive and HER‑2 negative metastatic breast cancer: A Bayesian network meta‑analysis
Source: Oncol Lett. 2024 Aug 28;28(5):513. doi: 10.3892/ol.2024.14646 (PMC11378012; doi:10.3892/ol.2024.14646)
Supplement: Supporting Data [file Supplementary_Data2.pdf]

Table SI. Matrix of the pairwise comparisons of regimens at 3 months of progression free survival (shown as odds ratio and 95% confidence intervals).

AbeAI	0.55<br>(0.05,5.64)	0.45<br>(0.10,2.05)	0.45<br>(0.09,2.40)	0.44<br>(0.06,3.20)	0.34<br>(0.05,2.25)	0.29<br>(0.05,1.64)	0.26<br>(0.03,1.99)	0.23<br>(0.04,1.27)	0.23<br>(0.05,1.19)	0.20<br>(0.05,0.78)
1.81<br>(0.18,18.49)	RiboFul	0.81<br>(0.11,6.13)	0.82<br>(0.10,6.90)	0.80<br>(0.11,5.97)	0.61<br>(0.06,6.21)	0.53<br>(0.06,4.68)	0.47<br>(0.04,5.34)	0.41<br>(0.05,3.63)	0.42<br>(0.08,2.22)	0.37<br>(0.06,2.45)
2.22<br>(0.49,10.12)	1.23<br>(0.16,9.24)	PalboAI	1.01<br>(0.31,3.32)	0.98<br>(0.20,4.92)	0.75<br>(0.16,3.40)	0.65<br>(0.18,2.34)	0.57<br>(0.14,2.25)	0.51<br>(0.14,1.81)	0.52<br>(0.16,1.63)	0.45<br>(0.22,0.91)
2.20<br>(0.42,11.65)	1.22<br>(0.14,10.22)	0.99<br>(0.30,3.26)	RiboAI	0.97<br>(0.17,5.58)	0.74<br>(0.14,3.91)	0.65<br>(0.15,2.76)	0.57<br>(0.09,3.48)	0.50<br>(0.12,2.14)	0.51<br>(0.14,1.94)	0.45<br>(0.17,1.19)
2.26<br>(0.31,16.39)	1.25<br>(0.17,9.33)	1.02<br>(0.20,5.10)	1.03<br>(0.18,5.89)	AbeFul	0.76<br>(0.11,5.51)	0.67<br>(0.11,4.04)	0.58<br>(0.07,4.83)	0.51<br>(0.08,3.13)	0.53<br>(0.17,1.64)	0.46<br>(0.11,1.97)
2.97<br>(0.44,19.83)	1.64<br>(0.16,16.71)	1.34<br>(0.29,6.07)	1.35<br>(0.26,7.10)	1.31<br>(0.18,9.48)	DalpAI	0.87<br>(0.16,4.84)	0.76<br>(0.10,5.88)	0.68<br>(0.12,3.75)	0.69<br>(0.14,3.51)	0.60<br>(0.16,2.30)
3.39<br>(0.61,18.85)	1.88<br>(0.21,16.48)	1.53<br>(0.43,5.46)	1.54<br>(0.36,6.56)	1.50<br>(0.25,9.09)	1.14<br>(0.21,6.33)	LapaAI	0.87<br>(0.13,5.67)	0.77<br>(0.17,3.49)	0.79<br>(0.20,3.22)	0.69<br>(0.24,2.00)
3.89<br>(0.50,30.09)	2.15<br>(0.19,24.68)	1.75<br>(0.44,6.91)	1.77<br>(0.29,10.87)	1.72<br>(0.21,14.28)	1.31<br>(0.17,10.11)	1.15<br>(0.18,7.46)	PalboFul	0.89<br>(0.14,5.78)	0.91<br>(0.15,5.43)	0.79<br>(0.17,3.69)
4.39<br>(0.79,24.48)	2.43<br>(0.28,21.38)	1.98<br>(0.55,7.10)	2.00<br>(0.47,8.52)	1.94<br>(0.32,11.80)	1.48<br>(0.27,8.22)	1.29<br>(0.29,5.84)	1.13<br>(0.17,7.37)	FulAI	1.03<br>(0.25,4.18)	0.89<br>(0.31,2.60)
4.29<br>(0.84,21.77)	2.37<br>(0.45,12.46)	1.93<br>(0.61,6.07)	1.95<br>(0.51,7.36)	1.89<br>(0.61,5.88)	1.44<br>(0.29,7.31)	1.26<br>(0.31,5.13)	1.10<br>(0.18,6.59)	0.98<br>(0.24,3.97)	Ful	0.87<br>(0.35,2.17)
4.92<br>(1.28,18.90)	2.72<br>(0.41,18.10)	2.22<br>(1.10,4.47)	2.24<br>(0.84,5.97)	2.18<br>(0.51,9.32)	1.66<br>(0.43,6.34)	1.45<br>(0.50,4.20)	1.27<br>(0.27,5.91)	1.12<br>(0.39,3.26)	1.15<br>(0.46,2.86)	AI

AI, aromatase inhibitor; AbeAI, abemaciclib plus AI; AbeFul, abemaciclib plus fulvestrant; DalpAI, dalpiciclib plus AI; Ful, fulvestrant; FulAI, fulvestrant plus AI; LapaAI, lapatinib plus AI; PalboAI, palbociclib plus AI; PalboFul, palbociclib plus fulvestrant; RiboAI, ribociclib plus AI; RiboFul, ribociclib plus fulvestrant.

Table SII. Matrix of the pairwise comparisons of regimens at 6 months of progression free survival (shown as odds ratio and 95% confidence intervals).

RiboFul	0.54 (0.09,3.24)	0.53 (0.09,3.27)	0.51 (0.07,3.75)	0.48 (0.07,3.11)	0.44 (0.06,3.29)	0.39 (0.05,3.29)	0.34 (0.08,1.46)	0.27 (0.04,1.88)	0.22 (0.03,1.56)	0.23 (0.04,1.19)
1.85 (0.31,11.13)	PalboAI	0.99 (0.22,4.50)	0.94 (0.25,3.48)	0.89 (0.30,2.63)	0.81 (0.21,3.07)	0.72 (0.22,2.30)	0.64 (0.22,1.83)	0.49 (0.14,1.70)	0.40 (0.12,1.41)	0.42 (0.21,0.82)
1.88 (0.31,11.51)	1.01 (0.22,4.61)	AbeFul	0.95 (0.16,5.52)	0.90 (0.18,4.49)	0.82 (0.14,4.85)	0.73 (0.11,4.92)	0.64 (0.22,1.92)	0.50 (0.09,2.75)	0.41 (0.07,2.28)	0.42 (0.11,1.65)
1.98 (0.27,14.68)	1.07 (0.29,3.96)	1.05 (0.18,6.14)	AbeAI	0.95 (0.23,3.90)	0.87 (0.17,4.30)	0.77 (0.13,4.42)	0.68 (0.17,2.71)	0.53 (0.11,2.42)	0.43 (0.09,2.01)	0.45 (0.15,1.38)
2.09 (0.32,13.58)	1.13 (0.38,3.34)	1.11 (0.22,5.57)	1.06 (0.26,4.36)	RiboAI	0.91 (0.22,3.84)	0.81 (0.16,3.98)	0.72 (0.22,2.34)	0.56 (0.14,2.14)	0.46 (0.12,1.78)	0.47 (0.20,1.12)
2.28 (0.30,17.18)	1.23 (0.33,4.66)	1.22 (0.21,7.20)	1.16 (0.23,5.75)	1.09 (0.26,4.59)	DalpAI	0.88 (0.15,5.18)	0.78 (0.19,3.19)	0.61 (0.13,2.85)	0.50 (0.11,2.37)	0.52 (0.16,1.62)
2.58 (0.30,21.90)	1.39 (0.43,4.47)	1.38 (0.20,9.32)	1.31 (0.23,7.54)	1.24 (0.25,6.08)	1.13 (0.19,6.62)	PalboFul	0.89 (0.18,4.27)	0.69 (0.13,3.75)	0.56 (0.10,3.12)	0.58 (0.15,2.25)
2.91 (0.68,12.41)	1.57 (0.55,4.51)	1.55 (0.52,4.62)	1.47 (0.37,5.88)	1.39 (0.43,4.55)	1.27 (0.31,5.18)	1.13 (0.23,5.43)	Ful	0.77 (0.21,2.88)	0.64 (0.17,2.40)	0.66 (0.29,1.48)
3.76 (0.53,26.66)	2.03 (0.59,7.01)	2.01 (0.36,11.08)	1.90 (0.41,8.78)	1.80 (0.47,6.95)	1.65 (0.35,7.72)	1.46 (0.27,7.98)	1.29 (0.35,4.82)	LapaAI	0.82 (0.19,3.60)	0.85 (0.30,2.40)
4.58 (0.64,32.77)	2.47 (0.71,8.66)	2.44 (0.44,13.63)	2.32 (0.50,10.82)	2.19 (0.56,8.58)	2.01 (0.42,9.51)	1.77 (0.32,9.82)	1.57 (0.42,5.95)	1.22 (0.28,5.34)	FulAI	1.04 (0.36,2.98)
4.42 (0.84,23.26)	2.39 (1.21,4.69)	2.36 (0.61,9.16)	2.23 (0.73,6.87)	2.12 (0.89,5.02)	1.93 (0.62,6.08)	1.71 (0.45,6.58)	1.52 (0.68,3.41)	1.17 (0.42,3.31)	0.96 (0.34,2.77)	AI

AI, aromatase inhibitor; AbeAI, abemaciclib plus AI; AbeFul, abemaciclib plus fulvestrant; DalpAI, dalpiciclib plus AI; Ful, fulvestrant; FulAI, fulvestrant plus AI; LapaAI, lapatinib plus AI; PalboAI, palbociclib plus AI; PalboFul, palbociclib plus fulvestrant; RiboAI, ribociclib plus AI; RiboFul, ribociclib plus fulvestrant.

Table SIII. Matrix of the pairwise comparisons of regimens at 12 months of progression free survival (shown as odds ratio and 95% confidence intervals).

RiboFul	0.85<br>(0.31,2.31)	0.67<br>(0.22,2.02)	0.66<br>(0.22,1.98)	0.63<br>(0.24,1.69)	0.63<br>(0.22,1.75)	0.57<br>(0.18,1.81)	0.44<br>(0.20,0.99)	0.32<br>(0.11,0.90)	0.31<br>(0.11,0.90)	0.33<br>(0.13,0.82)
1.18<br>(0.43,3.21)	AbeFul	0.79<br>(0.31,2.06)	0.78<br>(0.30,2.01)	0.74<br>(0.33,1.68)	0.74<br>(0.31,1.75)	0.67<br>(0.24,1.86)	0.52<br>(0.29,0.94)	0.38<br>(0.16,0.91)	0.37<br>(0.15,0.91)	0.38<br>(0.18,0.80)
1.49<br>(0.49,4.47)	1.26<br>(0.49,3.27)	DalpAI	0.98<br>(0.42,2.30)	0.94<br>(0.46,1.90)	0.93<br>(0.43,2.00)	0.85<br>(0.34,2.15)	0.66<br>(0.31,1.39)	0.47<br>(0.22,1.03)	0.46<br>(0.21,1.04)	0.48<br>(0.26,0.89)
1.52<br>(0.51,4.56)	1.29<br>(0.50,3.33)	1.02<br>(0.44,2.40)	AbeAI	0.96<br>(0.47,1.93)	0.95<br>(0.44,2.04)	0.87<br>(0.34,2.19)	0.67<br>(0.32,1.42)	0.48<br>(0.22,1.05)	0.47<br>(0.21,1.06)	0.49<br>(0.27,0.90)
1.59<br>(0.59,4.25)	1.35<br>(0.59,3.04)	1.07<br>(0.53,2.16)	1.05<br>(0.52,2.11)	PalboAI	0.99<br>(0.56,1.77)	0.91<br>(0.50,1.65)	0.70<br>(0.40,1.24)	0.51<br>(0.28,0.93)	0.49<br>(0.26,0.95)	0.52<br>(0.36,0.74)
1.59<br>(0.57,4.44)	1.35<br>(0.57,3.21)	1.07<br>(0.50,2.30)	1.05<br>(0.49,2.25)	1.01<br>(0.57,1.79)	RiboAI	0.91<br>(0.40,2.09)	0.71<br>(0.38,1.33)	0.51<br>(0.26,1.00)	0.50<br>(0.24,1.01)	0.52<br>(0.33,0.83)
1.75<br>(0.55,5.56)	1.49<br>(0.54,4.10)	1.18<br>(0.47,2.98)	1.15<br>(0.46,2.91)	1.10<br>(0.60,2.02)	1.10<br>(0.48,2.53)	PalboFul	0.78<br>(0.34,1.77)	0.56<br>(0.24,1.32)	0.54<br>(0.22,1.32)	0.57<br>(0.28,1.15)
2.26<br>(1.01,5.06)	1.91<br>(1.06,3.46)	1.52<br>(0.72,3.21)	1.49<br>(0.71,3.13)	1.42<br>(0.81,2.50)	1.41<br>(0.75,2.66)	1.29<br>(0.56,2.94)	Ful	0.72<br>(0.37,1.39)	0.70<br>(0.35,1.41)	0.73<br>(0.47,1.14)
3.13<br>(1.11,8.87)	2.66<br>(1.10,6.43)	2.11<br>(0.97,4.59)	2.06<br>(0.95,4.48)	1.98<br>(1.07,3.63)	1.96<br>(1.00,3.85)	1.79<br>(0.76,4.21)	1.39<br>(0.72,2.68)	LapaAI	0.97<br>(0.47,2.02)	1.02<br>(0.63,1.66)
3.22<br>(1.11,9.35)	2.73<br>(1.09,6.80)	2.16<br>(0.96,4.87)	2.12<br>(0.94,4.76)	2.03<br>(1.06,3.89)	2.02<br>(0.99,4.12)	1.84<br>(0.76,4.46)	1.43<br>(0.71,2.86)	1.03<br>(0.50,2.13)	FulAI	1.05<br>(0.61,1.80)
3.07<br>(1.22,7.71)	2.61<br>(1.25,5.44)	2.07<br>(1.13,3.79)	2.02<br>(1.11,3.70)	1.94<br>(1.34,2.79)	1.93<br>(1.21,3.07)	1.75<br>(0.87,3.55)	1.36<br>(0.88,2.11)	0.98<br>(0.60,1.60)	0.96<br>(0.56,1.64)	AI

AI, aromatase inhibitor; AbeAI, abemaciclib plus AI; AbeFul, abemaciclib plus fulvestrant; DalpAI, dalpiciclib plus AI; Ful, fulvestrant; FulAI, fulvestrant plus AI; LapaAI, lapatinib plus AI; PalboAI, palbociclib plus AI; PalboFul, palbociclib plus fulvestrant; RiboAI, ribociclib plus AI; RiboFul, ribociclib plus fulvestrant.

Table SIV. Matrix of the pairwise comparisons of regimens at 18 months of progression free survival (shown as odds ratio and 95% confidence intervals).

AbeFul	1.01 (0.41,2.50)	0.90 (0.39,2.06)	0.81 (0.30,2.20)	0.80 (0.31,2.06)	0.79 (0.31,2.04)	0.70 (0.29,1.68)	0.58 (0.31,1.06)	0.40 (0.16,1.03)	0.38 (0.18,0.80)	0.36 (0.15,0.88)
0.99 (0.40,2.46)	RiboFul	0.89 (0.37,2.16)	0.80 (0.28,2.28)	0.79 (0.29,2.14)	0.79 (0.29,2.12)	0.70 (0.28,1.75)	0.57 (0.29,1.13)	0.40 (0.15,1.07)	0.38 (0.17,0.84)	0.35 (0.14,0.91)
1.11 (0.48,2.54)	1.12 (0.46,2.70)	PalboAI	0.89 (0.51,1.58)	0.89 (0.45,1.76)	0.88 (0.45,1.75)	0.78 (0.43,1.40)	0.64 (0.36,1.13)	0.45 (0.23,0.88)	0.42 (0.29,0.61)	0.40 (0.21,0.73)
1.24 (0.45,3.38)	1.25 (0.44,3.56)	1.12 (0.63,1.97)	PalboFul	0.99 (0.41,2.41)	0.99 (0.41,2.39)	0.87 (0.38,1.97)	0.72 (0.32,1.59)	0.50 (0.21,1.21)	0.47 (0.24,0.92)	0.44 (0.19,1.02)
1.25 (0.49,3.24)	1.26 (0.47,3.42)	1.13 (0.57,2.25)	1.01 (0.42,2.46)	DalpAI	1.00 (0.44,2.26)	0.88 (0.43,1.81)	0.72 (0.35,1.50)	0.51 (0.22,1.14)	0.47 (0.27,0.85)	0.45 (0.21,0.96)
1.26 (0.49,3.24)	1.27 (0.47,3.42)	1.13 (0.57,2.24)	1.01 (0.42,2.46)	1.00 (0.44,2.27)	AbeAI	0.88 (0.43,1.81)	0.73 (0.35,1.50)	0.51 (0.23,1.14)	0.48 (0.27,0.85)	0.45 (0.21,0.96)
1.43 (0.60,3.42)	1.44 (0.57,3.62)	1.29 (0.71,2.32)	1.15 (0.51,2.60)	1.14 (0.55,2.35)	1.13 (0.55,2.33)	RiboAI	0.82 (0.44,1.54)	0.58 (0.28,1.17)	0.54 (0.35,0.83)	0.51 (0.26,0.98)
1.73 (0.94,3.18)	1.75 (0.89,3.43)	1.56 (0.89,2.75)	1.40 (0.63,3.11)	1.38 (0.67,2.86)	1.38 (0.67,2.84)	1.21 (0.65,2.27)	Ful	0.70 (0.34,1.43)	0.66 (0.42,1.02)	0.62 (0.32,1.20)
2.48 (0.97,6.34)	2.50 (0.93,6.69)	2.23 (1.14,4.38)	2.00 (0.83,4.82)	1.98 (0.88,4.45)	1.97 (0.88,4.41)	1.74 (0.85,3.54)	1.43 (0.70,2.93)	FulAI	0.94 (0.53,1.65)	0.89 (0.42,1.88)
2.64 (1.25,5.59)	2.66 (1.19,5.96)	2.38 (1.65,3.44)	2.13 (1.08,4.18)	2.11 (1.18,3.76)	2.10 (1.18,3.73)	1.85 (1.20,2.85)	1.52 (0.98,2.37)	1.07 (0.60,1.88)	AI	0.94 (0.58,1.55)
2.80 (1.14,6.86)	2.82 (1.09,7.26)	2.52 (1.36,4.66)	2.25 (0.98,5.20)	2.23 (1.04,4.78)	2.22 (1.04,4.74)	1.96 (1.02,3.78)	1.61 (0.83,3.13)	1.13 (0.53,2.39)	1.06 (0.65,1.74)	LapaAI

AI, aromatase inhibitor; AbeAI, abemaciclib plus AI; AbeFul, abemaciclib plus fulvestrant; DalpAI, dalpiciclib plus AI; Ful, fulvestrant; FulAI, fulvestrant plus AI; LapaAI, lapatinib plus AI; PalboAI, palbociclib plus AI; PalboFul, palbociclib plus fulvestrant; RiboAI, ribociclib plus AI; RiboFul, ribociclib plus fulvestrant.

Table SV. Matrix of the pairwise comparisons of regimens at 24 months of progression free survival (shown as odds ratio and 95% confidence intervals).

AbeFul	0.60 (0.27,1.34)	0.59 (0.24,1.46)	0.54 (0.21,1.40)	0.53 (0.23,1.24)	0.46 (0.20,1.05)	0.42 (0.23,0.77)	0.32 (0.13,0.80)	0.24 (0.10,0.57)	0.25 (0.12,0.52)
1.67 (0.74,3.77)	PalboAI	0.99 (0.52,1.88)	0.90 (0.54,1.50)	0.89 (0.40,1.99)	0.77 (0.45,1.32)	0.70 (0.41,1.21)	0.53 (0.28,1.03)	0.41 (0.23,0.73)	0.42 (0.29,0.60)
1.69 (0.69,4.18)	1.01 (0.53,1.93)	DalpAI	0.91 (0.40,2.07)	0.90 (0.37,2.21)	0.78 (0.41,1.50)	0.71 (0.36,1.40)	0.54 (0.25,1.16)	0.41 (0.21,0.83)	0.42 (0.25,0.72)
1.85 (0.71,4.83)	1.11 (0.67,1.84)	1.09 (0.48,2.48)	PalboFul	0.98 (0.38,2.55)	0.85 (0.41,1.79)	0.78 (0.37,1.64)	0.59 (0.26,1.36)	0.45 (0.21,0.97)	0.46 (0.25,0.86)
1.89 (0.81,4.39)	1.13 (0.50,2.52)	1.11 (0.45,2.74)	1.02 (0.39,2.64)	RiboFul	0.87 (0.38,1.96)	0.79 (0.44,1.43)	0.60 (0.24,1.49)	0.46 (0.20,1.08)	0.47 (0.23,0.97)
2.17 (0.96,4.94)	1.30 (0.76,2.23)	1.28 (0.67,2.46)	1.17 (0.56,2.46)	1.15 (0.51,2.61)	RiboAI	0.91 (0.52,1.60)	0.69 (0.36,1.35)	0.53 (0.30,0.95)	0.54 (0.37,0.79)
2.38 (1.31,4.35)	1.42 (0.83,2.46)	1.41 (0.72,2.76)	1.29 (0.61,2.71)	1.26 (0.70,2.29)	1.10 (0.63,1.92)	Ful	0.76 (0.38,1.51)	0.58 (0.32,1.07)	0.60 (0.39,0.90)
3.14 (1.26,7.84)	1.88 (0.97,3.63)	1.85 (0.86,4.00)	1.69 (0.74,3.89)	1.66 (0.67,4.14)	1.44 (0.74,2.81)	1.32 (0.66,2.62)	FulAI	0.77 (0.38,1.56)	0.78 (0.45,1.36)
4.09 (1.74,9.64)	2.45 (1.38,4.34)	2.42 (1.20,4.85)	2.21 (1.03,4.75)	2.17 (0.93,5.09)	1.88 (1.05,3.38)	1.72 (0.93,3.16)	1.30 (0.64,2.66)	LapaAI	1.02 (0.65,1.60)
4.01 (1.93,8.31)	2.39 (1.67,3.43)	2.36 (1.39,4.03)	2.16 (1.16,4.03)	2.12 (1.03,4.38)	1.84 (1.27,2.68)	1.68 (1.11,2.54)	1.28 (0.73,2.22)	0.98 (0.63,1.53)	AI

AI, aromatase inhibitor; AbeAI, abemaciclib plus AI; AbeFul, abemaciclib plus fulvestrant; DalpAI, dalpiciclib plus AI; Ful, fulvestrant; FulAI, fulvestrant plus AI; LapaAI, lapatinib plus AI; PalboAI, palbociclib plus AI; PalboFul, palbociclib plus fulvestrant; RiboAI, ribociclib plus AI; RiboFul, ribociclib plus fulvestrant.

Table SVI. Matrix of the pairwise comparisons of regimens at 30 months of progression free survival (shown as odds ratio and 95% confidence intervals).

AbeFul	0.80 (0.37,1.73)	0.47 (0.24,0.95)	0.43 (0.21,0.85)	0.36 (0.17,0.78)	0.33 (0.19,0.56)	0.32 (0.16,0.64)	0.26 (0.12,0.58)	0.26 (0.13,0.53)	0.20 (0.11,0.38)
1.25 (0.58,2.68)	DalpAI	0.59 (0.29,1.19)	0.53 (0.32,0.87)	0.45 (0.24,0.83)	0.41 (0.24,0.70)	0.40 (0.24,0.66)	0.32 (0.17,0.62)	0.33 (0.19,0.55)	0.25 (0.16,0.39)
2.11 (1.05,4.24)	1.69 (0.84,3.42)	RiboFul	0.90 (0.48,1.66)	0.76 (0.37,1.55)	0.69 (0.44,1.08)	0.68 (0.37,1.26)	0.54 (0.26,1.15)	0.55 (0.29,1.04)	0.43 (0.24,0.75)
2.35 (1.18,4.68)	1.89 (1.14,3.12)	1.12 (0.60,2.07)	PalboAI	0.85 (0.59,1.21)	0.77 (0.50,1.18)	0.76 (0.53,1.10)	0.61 (0.35,1.06)	0.62 (0.42,0.92)	0.48 (0.37,0.62)
2.77 (1.28,6.02)	2.23 (1.20,4.12)	1.32 (0.64,2.69)	1.18 (0.83,1.68)	PalboFul	0.91 (0.52,1.58)	0.90 (0.54,1.49)	0.72 (0.37,1.39)	0.73 (0.43,1.24)	0.56 (0.36,0.87)
3.05 (1.78,5.23)	2.45 (1.42,4.23)	1.45 (0.93,2.26)	1.30 (0.85,1.99)	1.10 (0.63,1.92)	Ful	0.99 (0.65,1.51)	0.79 (0.43,1.44)	0.80 (0.51,1.26)	0.62 (0.44,0.87)
3.09 (1.56,6.14)	2.48 (1.51,4.08)	1.47 (0.79,2.71)	1.32 (0.91,1.90)	1.12 (0.67,1.86)	1.01 (0.66,1.55)	RiboAI	0.80 (0.46,1.39)	0.81 (0.55,1.20)	0.63 (0.48,0.81)
3.87 (1.73,8.67)	3.11 (1.62,5.98)	1.84 (0.87,3.88)	1.65 (0.94,2.88)	1.40 (0.72,2.71)	1.27 (0.70,2.31)	1.25 (0.72,2.18)	FulAI	1.02 (0.57,1.81)	0.78 (0.48,1.28)
3.81 (1.89,7.67)	3.06 (1.82,5.14)	1.81 (0.96,3.40)	1.62 (1.09,2.41)	1.37 (0.81,2.34)	1.25 (0.79,1.95)	1.23 (0.83,1.82)	0.98 (0.55,1.75)	LapaAI	0.77 (0.57,1.04)
4.94 (2.62,9.33)	3.97 (2.59,6.08)	2.35 (1.34,4.10)	2.10 (1.62,2.74)	1.78 (1.15,2.78)	1.62 (1.15,2.27)	1.60 (1.24,2.06)	1.28 (0.78,2.09)	1.30 (0.97,1.75)	AI

AI, aromatase inhibitor; AbeAI, abemaciclib plus AI; AbeFul, abemaciclib plus fulvestrant; DalpAI, dalpiciclib plus AI; Ful, fulvestrant; FulAI, fulvestrant plus AI; LapaAI, lapatinib plus AI; PalboAI, palbociclib plus AI; PalboFul, palbociclib plus fulvestrant; RiboAI, ribociclib plus AI; RiboFul, ribociclib plus fulvestrant.

Table SVII. Matrix of the pairwise comparisons of regimens at 36 months of progression free survival (shown as odds ratio and 95% confidence intervals).

AbeFul	0.47 (0.11,2.00)	0.43 (0.10,1.82)	0.39 (0.07,2.22)	0.27 (0.09,0.79)	0.20 (0.04,1.01)	0.20 (0.04,1.01)	0.16 (0.04,0.59)	0.13 (0.02,0.71)
2.13 (0.50,9.05)	RiboFul	0.91 (0.23,3.65)	0.84 (0.16,4.51)	0.58 (0.22,1.56)	0.44 (0.09,2.04)	0.43 (0.09,2.05)	0.34 (0.10,1.17)	0.28 (0.06,1.43)
2.33 (0.55,9.91)	1.10 (0.27,4.39)	PalboAI	0.92 (0.35,2.38)	0.64 (0.24,1.70)	0.48 (0.15,1.52)	0.48 (0.15,1.52)	0.38 (0.19,0.73)	0.31 (0.09,1.09)
2.54 (0.45,14.33)	1.19 (0.22,6.41)	1.09 (0.42,2.82)	PalboFul	0.70 (0.18,2.72)	0.52 (0.12,2.32)	0.52 (0.11,2.33)	0.41 (0.13,1.31)	0.34 (0.07,1.63)
3.65 (1.26,10.58)	1.72 (0.64,4.58)	1.56 (0.59,4.16)	1.44 (0.37,5.63)	Ful	0.75 (0.23,2.46)	0.74 (0.22,2.48)	0.59 (0.28,1.22)	0.48 (0.13,1.76)
4.89 (0.99,24.16)	2.30 (0.49,10.77)	2.09 (0.66,6.64)	1.92 (0.43,8.59)	1.34 (0.41,4.41)	LapaAI	1.00 (0.26,3.81)	0.79 (0.31,2.02)	0.65 (0.16,2.69)
4.91 (0.99,24.44)	2.31 (0.49,10.90)	2.10 (0.66,6.74)	1.93 (0.43,8.70)	1.34 (0.40,4.47)	1.00 (0.26,3.85)	RiboAI	0.79 (0.30,2.05)	0.65 (0.16,2.72)
6.21 (1.71,22.57)	2.92 (0.86,9.93)	2.66 (1.37,5.18)	2.45 (0.77,7.81)	1.70 (0.82,3.53)	1.27 (0.49,3.26)	1.27 (0.49,3.29)	AI	0.82 (0.28,2.39)
7.53 (1.41,40.10)	3.54 (0.70,17.92)	3.23 (0.92,11.32)	2.97 (0.61,14.33)	2.06 (0.57,7.50)	1.54 (0.37,6.39)	1.53 (0.37,6.41)	1.21 (0.42,3.51)	FulAI

AI, aromatase inhibitor; AbeAI, abemaciclib plus AI; AbeFul, abemaciclib plus fulvestrant; DalpAI, dalpiciclib plus AI; Ful, fulvestrant; FulAI, fulvestrant plus AI; LapaAI, lapatinib plus AI; PalboAI, palbociclib plus AI; PalboFul, palbociclib plus fulvestrant; RiboAI, ribociclib plus AI; RiboFul, ribociclib plus fulvestrant.

Table SVIII. Matrix of the pairwise comparisons of regimens on hazard ratio of the progression free survival (shown as hazard ratio and 95% confidence intervals).

	AbeAI	AbeFul	AI	DalpAI	Ful	FulAI	LapaAI	PalboAI	PalboFul	RiboAI	RiboFul
AbeAI	AbeAI	1.25 (0.67-2.43)	0.51 (0.32-0.8)	1 (0.54-1.85)	0.71 (0.43-1.21)	0.52 (0.29-0.92)	0.57 (0.32-1)	0.87 (0.52-1.45)	1.5 (0.73-3.1)	0.9 (0.53-1.52)	1.14 (0.6-2.23)
AbeFul	0.8 (0.41-1.49)	AbeFul	0.41 (0.25-0.63)	0.8 (0.43-1.46)	0.57 (0.39-0.83)	0.41 (0.23-0.72)	0.45 (0.25-0.78)	0.69 (0.41-1.14)	1.19 (0.63-2.25)	0.72 (0.42-1.19)	0.91 (0.52-1.59)
AI	1.96 (1.25-3.09)	2.45 (1.59-3.93)	AI	1.96 (1.29-2.98)	1.4 (1.11-1.82)	1.01 (0.71-1.44)	1.11 (0.79-1.56)	1.7 (1.36-2.16)	2.93 (1.68-5.21)	1.76 (1.35-2.3)	2.23 (1.4-3.65)
DalpAI	1 (0.54-1.85)	1.25 (0.69-2.35)	0.51 (0.34-0.77)	DalpAI	0.71 (0.45-1.17)	0.52 (0.3-0.89)	0.57 (0.33-0.97)	0.87 (0.54-1.41)	1.49 (0.75-3.05)	0.9 (0.55-1.48)	1.14 (0.61-2.18)
Ful	1.4 (0.82-2.31)	1.75 (1.2-2.56)	0.71 (0.55-0.9)	1.4 (0.85-2.24)	Ful	0.72 (0.46-1.09)	0.79 (0.51-1.18)	1.21 (0.86-1.69)	2.09 (1.25-3.47)	1.26 (0.86-1.78)	1.59 (1.05-2.4)
FulAI	1.94 (1.09-3.45)	2.43 (1.4-4.4)	0.99 (0.69-1.41)	1.94 (1.12-3.35)	1.39 (0.92-2.17)	FulAI	1.1 (0.67-1.79)	1.68 (1.11-2.6)	2.9 (1.51-5.68)	1.74 (1.12-2.72)	2.2 (1.24-4.08)
LapaAI	1.76 (1-3.11)	2.21 (1.28-3.97)	0.9 (0.64-1.26)	1.76 (1.03-3.01)	1.26 (0.85-1.94)	0.91 (0.56-1.48)	LapaAI	1.53 (1.03-2.33)	2.63 (1.38-5.14)	1.58 (1.03-2.44)	2 (1.14-3.65)
PalboAI	1.15 (0.69-1.91)	1.45 (0.88-2.42)	0.59 (0.46-0.73)	1.15 (0.71-1.85)	0.82 (0.59-1.16)	0.59 (0.38-0.9)	0.65 (0.43-0.97)	PalboAI	1.72 (0.94-3.18)	1.04 (0.72-1.46)	1.31 (0.78-2.24)
PalboFul	0.67 (0.32-1.36)	0.84 (0.44-1.59)	0.34 (0.19-0.6)	0.67 (0.33-1.33)	0.48 (0.29-0.8)	0.34 (0.18-0.66)	0.38 (0.19-0.72)	0.58 (0.31-1.06)	PalboFul	0.6 (0.32-1.11)	0.6 (0.32-1.12)
RiboAI	1.11 (0.66-1.89)	1.39 (0.84-2.4)	0.57 (0.44-0.74)	1.11 (0.68-1.83)	0.8 (0.56-1.16)	0.57 (0.37-0.9)	0.63 (0.41-0.97)	0.97 (0.68-1.39)	1.66 (0.9-3.14)	RiboAI	1.26 (0.74-2.22)
RiboFul	0.88 (0.45-1.68)	1.1 (0.63-1.93)	0.45 (0.27-0.71)	0.88 (0.46-1.64)	0.63 (0.42-0.95)	0.45 (0.25-0.81)	0.5 (0.27-0.88)	0.76 (0.45-1.29)	1.31 (0.69-2.52)	0.79 (0.45-1.35)	RiboFul

AI, aromatase inhibitor; AbeAI, abemaciclib plus AI; AbeFul, abemaciclib plus fulvestrant; DalpAI, dalpiciclib plus AI; Ful, fulvestrant; FulAI, fulvestrant plus AI; LapaAI, lapatinib plus AI; PalboAI, palbociclib plus AI; PalboFul, palbociclib plus fulvestrant; RiboAI, ribociclib plus AI; RiboFul, ribociclib plus fulvestrant

Table SIX. Matrix of the pairwise comparisons of SMD for regimens on the absolute progression-free survival value.

PalboFul	-0.25 (-0.61,0.11)	-0.27 (-0.64,0.11)	-0.50 (-0.79,-0.21)	0.38 (-0.03,0.78)
0.25 (-0.11,0.61)	PalboAI	-0.02 (-0.21,0.18)	-0.25 (-0.47,-0.03)	-0.37 (-0.49,-0.25)
0.27 (-0.11,0.64)	0.02 (-0.18,0.21)	RiboAI	-0.23 (-0.47,0.01)	-0.62 (-0.96,-0.28)
0.50 (0.21,0.79)	0.25 (0.03,0.47)	0.23 (-0.01,0.47)	Ful	-0.12 (-0.30,0.06)
-0.38 (-0.78,0.03)	0.37 (0.25,0.49)	0.62 (0.28,0.96)	0.12 (-0.06,0.30)	AI

SMD, standard mean difference; AI, aromatase inhibitor; Ful, fulvestrant; PalboAI, palbociclib plus AI; PalboFul, palbociclib plus fulvestrant; RiboAI, ribociclib plus AI.

Table SX. Matrix of the pairwise comparisons of regimens at 3 months overall survival (shown as odds ratio and 95% confidence intervals).

AbeFul	0.93 (0.11,7.88)	0.67 (0.02,27.77)	0.66 (0.15,3.01)	0.34 (0.01,18.58)	0.38 (0.02,7.68)	0.33 (0.02,5.75)	0.27 (0.00,18.35)
1.08 (0.13,9.14)	RiboFul	0.72 (0.02,29.92)	0.72 (0.16,3.25)	0.37 (0.01,20.02)	0.41 (0.02,8.27)	0.36 (0.02,6.19)	0.30 (0.00,19.77)
1.50 (0.04,62.57)	1.39 (0.03,58.12)	RiboAI	1.00 (0.03,30.17)	0.52 (0.01,20.58)	0.57 (0.04,7.59)	0.50 (0.04,5.52)	0.41 (0.01,20.68)
1.51 (0.33,6.83)	1.40 (0.31,6.35)	1.00 (0.03,30.36)	Ful	0.52 (0.01,20.77)	0.57 (0.04,7.69)	0.50 (0.04,5.61)	0.41 (0.01,20.86)
2.90 (0.05,156.50)	2.69 (0.05,145.35)	1.93 (0.05,76.95)	1.93 (0.05,77.18)	PalboAI	1.10 (0.06,21.04)	0.96 (0.06,15.70)	0.80 (0.21,3.00)
2.64 (0.13,53.70)	2.46 (0.12,49.88)	1.76 (0.13,23.55)	1.76 (0.13,23.73)	0.91 (0.05,17.47)	FulAI	0.88 (0.33,2.31)	0.73 (0.03,18.50)
3.01 (0.17,52.12)	2.80 (0.16,48.42)	2.01 (0.18,22.23)	2.00 (0.18,22.43)	1.04 (0.06,16.90)	1.14 (0.43,3.00)	AI	0.83 (0.04,18.17)
3.64 (0.05,243.58)	3.38 (0.05,226.23)	2.43 (0.05,121.81)	2.42 (0.05,122.11)	1.26 (0.33,4.73)	1.38 (0.05,35.11)	1.21 (0.06,26.60)	PalboFul

AI, aromatase inhibitor; AbeAI, abemaciclib plus AI; AbeFul, abemaciclib plus fulvestrant; DalpAI, dalpiciclib plus AI; Ful, fulvestrant; FulAI, fulvestrant plus AI; LapaAI, lapatinib plus AI; PalboAI, palbociclib plus AI; PalboFul, palbociclib plus fulvestrant; RiboAI, ribociclib plus AI; RiboFul, ribociclib plus fulvestrant.

Table SXI. Matrix of the pairwise comparisons of regimens at 6 months of overall survival (shown as odds ratio and 95% confidence intervals).

AbeFul	0.54 (0.10,3.05)	0.48 (0.19,1.25)	0.38 (0.04,3.84)	0.35 (0.05,2.31)	0.29 (0.05,1.64)	0.07 (0.00,1.20)	0.04 (0.00,0.92)
1.85 (0.33,10.47)	RiboFul	0.90 (0.21,3.82)	0.71 (0.06,9.11)	0.65 (0.07,5.75)	0.53 (0.07,4.17)	0.13 (0.01,2.73)	0.08 (0.00,2.07)
2.06 (0.80,5.33)	1.11 (0.26,4.74)	Ful	0.79 (0.10,6.46)	0.73 (0.14,3.68)	0.59 (0.14,2.56)	0.15 (0.01,2.10)	0.09 (0.01,1.64)
2.60 (0.26,25.94)	1.40 (0.11,17.91)	1.26 (0.15,10.24)	RiboAI	0.92 (0.17,4.83)	0.75 (0.17,3.37)	0.19 (0.01,2.72)	0.12 (0.01,2.11)
2.84 (0.43,18.57)	1.53 (0.17,13.45)	1.37 (0.27,6.95)	1.09 (0.21,5.76)	FulAI	0.82 (0.40,1.66)	0.20 (0.02,2.09)	0.13 (0.01,1.67)
3.48 (0.61,19.81)	1.87 (0.24,14.65)	1.68 (0.39,7.24)	1.34 (0.30,6.02)	1.23 (0.60,2.49)	AI	0.25 (0.03,2.29)	0.15 (0.01,1.85)
13.90 (0.83,232.05)	7.50 (0.37,153.65)	6.73 (0.48,95.34)	5.35 (0.37,77.76)	4.90 (0.48,50.07)	4.00 (0.44,36.60)	PalboAI	0.62 (0.20,1.91)
22.53 (1.08,468.10)	12.15 (0.48,305.68)	10.91 (0.61,194.76)	8.67 (0.47,158.52)	7.94 (0.60,105.34)	6.48 (0.54,77.91)	1.62 (0.52,5.03)	PalboFul

AI, aromatase inhibitor; AbeAI, abemaciclib plus AI; AbeFul, abemaciclib plus fulvestrant; DalpAI, dalpiciclib plus AI; Ful, fulvestrant; FulAI, fulvestrant plus AI; LapaAI, lapatinib plus AI; PalboAI, palbociclib plus AI; PalboFul, palbociclib plus fulvestrant; RiboAI, ribociclib plus AI; RiboFul, ribociclib plus fulvestrant.

Table SXII. Matrix of the pairwise comparisons of regimens at 12 months of overall survival (shown as odds ratio and 95% confidence intervals).

AbeFul	0.91 (0.29,2.84)	0.76 (0.16,3.66)	0.62 (0.16,2.30)	0.61 (0.15,2.52)	0.64 (0.32,1.30)	0.57 (0.10,3.25)	0.57 (0.17,1.93)
1.10 (0.35,3.42)	RiboFul	0.84 (0.16,4.41)	0.68 (0.16,2.82)	0.67 (0.15,3.06)	0.71 (0.29,1.72)	0.63 (0.10,3.89)	0.63 (0.16,2.39)
1.31 (0.27,6.27)	1.19 (0.23,6.27)	PalboAI	0.81 (0.27,2.44)	0.80 (0.24,2.71)	0.84 (0.21,3.41)	0.75 (0.36,1.58)	0.75 (0.28,2.00)
1.62 (0.43,6.08)	1.48 (0.35,6.17)	1.24 (0.41,3.75)	FulAI	0.99 (0.41,2.39)	1.04 (0.34,3.18)	0.93 (0.25,3.54)	0.93 (0.56,1.54)
1.64 (0.40,6.75)	1.49 (0.33,6.81)	1.25 (0.37,4.24)	1.01 (0.42,2.43)	RiboAI	1.05 (0.31,3.59)	0.94 (0.22,3.93)	0.93 (0.45,1.92)
1.56 (0.77,3.15)	1.42 (0.58,3.46)	1.19 (0.29,4.82)	0.96 (0.31,2.92)	0.95 (0.28,3.25)	Ful	0.89 (0.18,4.36)	0.89 (0.33,2.40)
1.74 (0.31,9.87)	1.59 (0.26,9.78)	1.33 (0.63,2.80)	1.07 (0.28,4.07)	1.06 (0.25,4.45)	1.12 (0.23,5.46)	PalboFul	0.99 (0.29,3.42)
1.75 (0.52,5.93)	1.60 (0.42,6.07)	1.34 (0.50,3.58)	1.08 (0.65,1.79)	1.07 (0.52,2.20)	1.12 (0.42,3.04)	1.01 (0.29,3.46)	AI

AI, aromatase inhibitor; AbeAI, abemaciclib plus AI; AbeFul, abemaciclib plus fulvestrant; DalpAI, dalpiciclib plus AI; Ful, fulvestrant; FulAI, fulvestrant plus AI; LapaAI, lapatinib plus AI; PalboAI, palbociclib plus AI; PalboFul, palbociclib plus fulvestrant; RiboAI, ribociclib plus AI; RiboFul, ribociclib plus fulvestrant.

Table SXIII. Matrix of the pairwise comparisons of regimens at 18 months of overall survival (shown as odds ratio and 95% confidence intervals).

RiboFul	0.56 (0.23,1.38)	0.51 (0.13,2.01)	0.47 (0.23,0.97)	0.36 (0.11,1.23)	0.37 (0.08,1.65)	0.33 (0.10,1.06)	0.31 (0.10,0.93)
1.78 (0.73,4.37)	AbeFul	0.90 (0.25,3.28)	0.84 (0.49,1.44)	0.65 (0.21,1.99)	0.66 (0.16,2.72)	0.59 (0.20,1.70)	0.55 (0.21,1.48)
1.97 (0.50,7.80)	1.11 (0.30,4.02)	PalboAI	0.93 (0.29,3.01)	0.72 (0.26,1.95)	0.73 (0.40,1.32)	0.65 (0.25,1.65)	0.61 (0.26,1.41)
2.12 (1.03,4.35)	1.19 (0.70,2.03)	1.07 (0.33,3.47)	Ful	0.77 (0.29,2.07)	0.78 (0.21,2.91)	0.69 (0.28,1.75)	0.66 (0.29,1.50)
2.75 (0.81,9.35)	1.55 (0.50,4.76)	1.40 (0.51,3.80)	1.30 (0.48,3.50)	RiboAI	1.01 (0.32,3.25)	0.90 (0.45,1.80)	0.86 (0.49,1.48)
2.71 (0.61,12.16)	1.52 (0.37,6.31)	1.38 (0.76,2.50)	1.28 (0.34,4.78)	0.99 (0.31,3.16)	PalboFul	0.89 (0.29,2.70)	0.84 (0.30,2.36)
3.04 (0.94,9.81)	1.71 (0.59,4.97)	1.55 (0.61,3.94)	1.44 (0.57,3.62)	1.11 (0.55,2.21)	1.12 (0.37,3.41)	FulAI	0.95 (0.62,1.44)
3.22 (1.08,9.60)	1.81 (0.68,4.82)	1.63 (0.71,3.77)	1.52 (0.67,3.46)	1.17 (0.68,2.02)	1.19 (0.42,3.32)	1.06 (0.70,1.61)	AI

AI, aromatase inhibitor; AbeAI, abemaciclib plus AI; AbeFul, abemaciclib plus fulvestrant; DalpAI, dalpiciclib plus AI; Ful, fulvestrant; FulAI, fulvestrant plus AI; LapaAI, lapatinib plus AI; PalboAI, palbociclib plus AI; PalboFul, palbociclib plus fulvestrant; RiboAI, ribociclib plus AI; RiboFul, ribociclib plus fulvestrant.

Table SXIV. Matrix of the pairwise comparisons of regimens at 24 months of overall survival (shown as odds ratio and 95% confidence intervals).

RiboFul	0.95 (0.45,1.98)	0.78 (0.25,2.43)	0.75 (0.21,2.63)	0.76 (0.44,1.34)	0.71 (0.27,1.85)	0.64 (0.24,1.71)	0.57 (0.23,1.38)
1.06 (0.51,2.21)	AbeFul	0.82 (0.27,2.47)	0.79 (0.23,2.68)	0.81 (0.50,1.30)	0.75 (0.30,1.87)	0.67 (0.26,1.73)	0.60 (0.26,1.39)
1.29 (0.41,4.03)	1.22 (0.40,3.67)	PalboAI	0.97 (0.57,1.63)	0.98 (0.36,2.65)	0.91 (0.40,2.04)	0.82 (0.36,1.89)	0.73 (0.36,1.50)
1.33 (0.38,4.68)	1.26 (0.37,4.27)	1.04 (0.61,1.75)	PalboFul	1.02 (0.33,3.12)	0.94 (0.36,2.47)	0.85 (0.32,2.27)	0.76 (0.31,1.84)
1.31 (0.75,2.30)	1.24 (0.77,2.00)	1.02 (0.38,2.75)	0.98 (0.32,3.02)	Ful	0.93 (0.42,2.03)	0.84 (0.37,1.88)	0.75 (0.37,1.48)
1.42 (0.54,3.72)	1.34 (0.53,3.37)	1.10 (0.49,2.47)	1.06 (0.41,2.79)	1.08 (0.49,2.37)	FulAI	0.91 (0.51,1.60)	0.81 (0.55,1.18)
1.57 (0.59,4.19)	1.48 (0.58,3.79)	1.22 (0.53,2.79)	1.17 (0.44,3.13)	1.19 (0.53,2.68)	1.10 (0.63,1.95)	RiboAI	0.89 (0.58,1.36)
1.76 (0.72,4.28)	1.66 (0.72,3.85)	1.37 (0.67,2.80)	1.32 (0.54,3.20)	1.34 (0.67,2.67)	1.24 (0.85,1.81)	1.12 (0.74,1.72)	AI

AI, aromatase inhibitor; AbeAI, abemaciclib plus AI; AbeFul, abemaciclib plus fulvestrant; DalpAI, dalpiciclib plus AI; Ful, fulvestrant; FulAI, fulvestrant plus AI; LapaAI, lapatinib plus AI; PalboAI, palbociclib plus AI; PalboFul, palbociclib plus fulvestrant; RiboAI, ribociclib plus AI; RiboFul, ribociclib plus fulvestrant.

Table SXV. Matrix of the pairwise comparisons of regimens at 24 months of overall survival (shown as odds ratio and 95% confidence intervals).

RiboFul	0.87 (0.45,1.68)	0.77 (0.32,1.84)	0.70 (0.23,2.11)	0.68 (0.25,1.86)	0.69 (0.42,1.13)	0.55 (0.25,1.20)	0.53 (0.22,1.25)
1.15 (0.60,2.22)	AbeFul	0.89 (0.38,2.05)	0.80 (0.27,2.38)	0.78 (0.29,2.08)	0.80 (0.51,1.23)	0.63 (0.29,1.34)	0.61 (0.26,1.40)
1.30 (0.54,3.11)	1.13 (0.49,2.62)	RiboAI	0.90 (0.38,2.14)	0.88 (0.42,1.82)	0.90 (0.44,1.85)	0.71 (0.49,1.02)	0.69 (0.41,1.14)
1.44 (0.47,4.37)	1.25 (0.42,3.71)	1.11 (0.47,2.62)	PalboFul	0.97 (0.61,1.55)	0.99 (0.37,2.70)	0.79 (0.36,1.72)	0.76 (0.32,1.79)
1.48 (0.54,4.06)	1.29 (0.48,3.44)	1.14 (0.55,2.35)	1.03 (0.65,1.64)	PalboAI	1.02 (0.42,2.47)	0.81 (0.43,1.52)	0.78 (0.38,1.60)
1.45 (0.89,2.36)	1.26 (0.81,1.95)	1.11 (0.54,2.28)	1.01 (0.37,2.73)	0.98 (0.40,2.36)	Ful	0.79 (0.42,1.47)	0.76 (0.37,1.56)
1.83 (0.83,4.04)	1.59 (0.74,3.40)	1.41 (0.98,2.03)	1.27 (0.58,2.78)	1.24 (0.66,2.32)	1.27 (0.68,2.35)	AI	0.96 (0.68,1.37)
1.90 (0.80,4.51)	1.65 (0.71,3.81)	1.46 (0.88,2.43)	1.32 (0.56,3.11)	1.28 (0.62,2.64)	1.31 (0.64,2.68)	1.04 (0.73,1.48)	FulAI

AI, aromatase inhibitor; AbeAI, abemaciclib plus AI; AbeFul, abemaciclib plus fulvestrant; DalpAI, dalpiciclib plus AI; Ful, fulvestrant; FulAI, fulvestrant plus AI; LapaAI, lapatinib plus AI; PalboAI, palbociclib plus AI; PalboFul, palbociclib plus fulvestrant; RiboAI, ribociclib plus AI; RiboFul, ribociclib plus fulvestrant.

Table SXVI. Matrix of the pairwise comparisons of regimens at 24 months of overall survival (shown as odds ratio and 95% confidence intervals).

RiboFul	0.85 (0.45,1.58)	0.69 (0.24,1.97)	0.60 (0.27,1.33)	0.63 (0.39,1.00)	0.59 (0.23,1.52)	0.50 (0.24,1.03)	0.47 (0.21,1.05)
1.18 (0.63,2.22)	AbeFul	0.82 (0.29,2.29)	0.71 (0.33,1.54)	0.74 (0.49,1.13)	0.69 (0.27,1.76)	0.59 (0.29,1.19)	0.55 (0.25,1.21)
1.44 (0.51,4.10)	1.22 (0.44,3.40)	PalboFul	0.86 (0.38,1.95)	0.90 (0.35,2.30)	0.85 (0.55,1.30)	0.72 (0.34,1.51)	0.67 (0.30,1.53)
1.67 (0.75,3.73)	1.41 (0.65,3.08)	1.16 (0.51,2.62)	RiboAI	1.05 (0.54,2.01)	0.98 (0.49,1.97)	0.83 (0.60,1.16)	0.78 (0.48,1.26)
1.60 (1.00,2.55)	1.35 (0.89,2.06)	1.11 (0.44,2.82)	0.96 (0.50,1.84)	Ful	0.94 (0.41,2.16)	0.79 (0.45,1.39)	0.75 (0.38,1.44)
1.70 (0.66,4.41)	1.44 (0.57,3.65)	1.18 (0.77,1.81)	1.02 (0.51,2.04)	1.06 (0.46,2.44)	PalboAI	0.85 (0.46,1.56)	0.79 (0.39,1.60)
2.01 (0.97,4.18)	1.70 (0.84,3.44)	1.40 (0.66,2.94)	1.20 (0.86,1.68)	1.26 (0.72,2.21)	1.18 (0.64,2.18)	AI	0.94 (0.66,1.33)
2.15 (0.96,4.82)	1.81 (0.83,3.97)	1.49 (0.65,3.39)	1.28 (0.79,2.07)	1.34 (0.69,2.60)	1.26 (0.62,2.55)	1.07 (0.75,1.51)	FulAI

AI, aromatase inhibitor; AbeAI, abemaciclib plus AI; AbeFul, abemaciclib plus fulvestrant; DalpAI, dalpiciclib plus AI; Ful, fulvestrant; FulAI, fulvestrant plus AI; LapaAI, lapatinib plus AI; PalboAI, palbociclib plus AI; PalboFul, palbociclib plus fulvestrant; RiboAI, ribociclib plus AI; RiboFul, ribociclib plus fulvestrant.

Table SXVII. Matrix of the pairwise comparisons of regimens on hazard ratio of the overall survival (shown as odds ratio and 95% confidence intervals).

	AbeAI	AbeFul	AI	Ful	FulAI	PalboAI	PalboFul	RiboAI	RiboFul
AbeAI	AbeAI	1.13 (0.58, 2.21)	0.76 (0.5, 1.13)	0.96 (0.57, 1.61)	0.76 (0.42, 1.35)	0.81 (0.49, 1.34)	0.81 (0.4, 1.63)	0.97 (0.59, 1.58)	1.5 (0.75, 2.98)
AbeFul	0.88 (0.45, 1.74)	AbeFul	0.67 (0.39, 1.14)	0.85 (0.56, 1.31)	0.67 (0.34, 1.32)	0.71 (0.39, 1.32)	0.71 (0.33, 1.57)	0.86 (0.47, 1.57)	1.32 (0.71, 2.46)
AI	1.32 (0.88, 1.99)	1.5 (0.88, 2.55)	AI	1.27 (0.92, 1.76)	1 (0.66, 1.52)	1.07 (0.8, 1.44)	1.07 (0.6, 1.9)	1.29 (0.98, 1.7)	1.99 (1.14, 3.47)
Ful	1.04 (0.62, 1.75)	1.18 (0.77, 1.8)	0.78 (0.57, 1.09)	Ful	0.79 (0.46, 1.33)	0.84 (0.54, 1.3)	0.84 (0.44, 1.63)	1.01 (0.66, 1.54)	1.56 (0.99, 2.45)
FulAI	1.32 (0.74, 2.37)	1.5 (0.76, 2.95)	1 (0.66, 1.52)	1.27 (0.75, 2.16)	FulAI	1.07 (0.64, 1.78)	1.07 (0.53, 2.17)	1.29 (0.78, 2.13)	1.99 (0.99, 3.99)
PalboAI	1.24 (0.75, 2.04)	1.41 (0.76, 2.57)	0.94 (0.69, 1.25)	1.19 (0.77, 1.84)	0.94 (0.56, 1.55)	PalboAI	1 (0.61, 1.64)	1.21 (0.8, 1.79)	1.86 (0.99, 3.47)
PalboFul	1.24 (0.61, 2.51)	1.4 (0.64, 3.07)	0.93 (0.53, 1.66)	1.19 (0.61, 2.29)	0.93 (0.46, 1.9)	1 (0.61, 1.64)	PalboFul	1.21 (0.64, 2.27)	1.86 (0.83, 4.12)
RiboAI	1.03 (0.63, 1.69)	1.16 (0.64, 2.13)	0.77 (0.59, 1.03)	0.99 (0.65, 1.51)	0.78 (0.47, 1.28)	0.83 (0.56, 1.25)	0.83 (0.44, 1.57)	RiboAI	1.54 (0.83, 2.88)
RiboFul	0.67 (0.34, 1.33)	0.76 (0.41, 1.4)	0.5 (0.29, 0.88)	0.64 (0.41, 1.01)	0.5 (0.25, 1.01)	0.54 (0.29, 1.01)	0.54 (0.24, 1.2)	0.65 (0.35, 1.2)	RiboFul

AI, aromatase inhibitor; AbeAI, abemaciclib plus AI; AbeFul, abemaciclib plus fulvestrant; DalpAI, dalpiciclib plus AI; Ful, fulvestrant; FulAI, fulvestrant plus AI; LapaAI, lapatinib plus AI; PalboAI, palbociclib plus AI; PalboFul, palbociclib plus fulvestrant; RiboAI, ribociclib plus AI; RiboFul, ribociclib plus fulvestrant.
